# Phenotypic, functional, prognostic and predictive significance of B-cell and antibody responses in human melanoma: a scoping review

**DOI:** 10.1093/bjd/ljag074

**Published:** 2026-02-26

**Authors:** Lucy Booth, Xinyi Chen, Katie Stoker, Alexandra McCraw, Mauzammal Tahiri, Yin Wu, Jenny L C Geh, Alastair D MacKenzie Ross, Hawys Lloyd-Hughes, Katie E Lacy, Sean Whittaker, Joanna Jacków-Malinowska, Mieke Van Hemelrijck, Thomas J Tull, Sophia Tsoka, Sophia N Karagiannis

**Affiliations:** St John’s Institute of Dermatology, School of Basic and Medical Biosciences and KHP Centre for Translational Medicine, Guy’s Hospital, King’s College London, London, UK; Department of Informatics, Faculty of Natural, Mathematical and Engineering Sciences, King’s College London, London, UK; St John’s Institute of Dermatology, School of Basic and Medical Biosciences and KHP Centre for Translational Medicine, Guy’s Hospital, King’s College London, London, UK; St John’s Institute of Dermatology, School of Basic and Medical Biosciences and KHP Centre for Translational Medicine, Guy’s Hospital, King’s College London, London, UK; Department of Informatics, Faculty of Natural, Mathematical and Engineering Sciences, King’s College London, London, UK; St John’s Institute of Dermatology, School of Basic and Medical Biosciences and KHP Centre for Translational Medicine, Guy’s Hospital, King’s College London, London, UK; St John’s Institute of Dermatology, School of Basic and Medical Biosciences and KHP Centre for Translational Medicine, Guy’s Hospital, King’s College London, London, UK; St John’s Institute of Dermatology, School of Basic and Medical Biosciences and KHP Centre for Translational Medicine, Guy’s Hospital, King’s College London, London, UK; Department of Medical Oncology, Guy’s and St Thomas’ Hospital, London, UK; Breast Cancer Now Research Unit, School of Cancer and Pharmaceutical Sciences, King’s College London, Innovation Hub, Guy’s Hospital, London, UK; Centre for Inflammation Biology and Cancer Immunology, School of Immunology and Microbial Sciences, King’s College London, London, UK; St John’s Institute of Dermatology, School of Basic and Medical Biosciences and KHP Centre for Translational Medicine, Guy’s Hospital, King’s College London, London, UK; St John’s Institute of Dermatology, Guy’s and St Thomas’ Hospitals NHS Foundation Trust, London, UK; Department of Plastic Surgery, Guy’s and St Thomas’ Hospitals, London, UK; Department of Plastic Surgery, Guy’s and St Thomas’ Hospitals, London, UK; Department of Plastic Surgery, Guy’s and St Thomas’ Hospitals, London, UK; St John’s Institute of Dermatology, School of Basic and Medical Biosciences and KHP Centre for Translational Medicine, Guy’s Hospital, King’s College London, London, UK; St John’s Institute of Dermatology, School of Basic and Medical Biosciences and KHP Centre for Translational Medicine, Guy’s Hospital, King’s College London, London, UK; St John’s Institute of Dermatology, School of Basic and Medical Biosciences and KHP Centre for Translational Medicine, Guy’s Hospital, King’s College London, London, UK; Translational Oncology and Urology Research (TOUR), School of Cancer and Pharmaceutical Sciences, King’s College, London, UK; St John’s Institute of Dermatology, School of Basic and Medical Biosciences and KHP Centre for Translational Medicine, Guy’s Hospital, King’s College London, London, UK; Department of Informatics, Faculty of Natural, Mathematical and Engineering Sciences, King’s College London, London, UK; St John’s Institute of Dermatology, School of Basic and Medical Biosciences and KHP Centre for Translational Medicine, Guy’s Hospital, King’s College London, London, UK; Breast Cancer Now Research Unit, School of Cancer and Pharmaceutical Sciences, King’s College London, Innovation Hub, Guy’s Hospital, London, UK

## Abstract

**Background:**

The clinical significance of B cells and the antibodies they express is increasingly appreciated in melanoma, a highly immunogenic tumour, for which immune checkpoint inhibitor (ICI) therapy is the standard care for advanced disease.

**Objectives:**

To evaluate the phenotypes and roles of B cells and antibodies in patients with melanoma, and their prognostic and predictive value, in a scoping review following PRISMA-ScR reporting guidelines.

**Methods:**

Using three search engines, we conducted literature searches for full-text studies written in English from 1 January 2000 to 9 October 2024. Three reviewers conducted title and abstract screening, followed by full-text paper assessment by two independent reviewers. This study was registered with PROSPERO (CRD42024592965).

**Results:**

Of 4667 identified studies (PubMed, *n* = 827; Scopus, *n* = 2759; Ovid MEDLINE, *n* = 1081), 1659 were duplicates. The titles and abstracts of the remaining 3008 were screened to yield 251 full-text papers, resulting in the inclusion of 80 studies. Our search identified increased naïve, alternatively activated and regulatory B cells in blood, and a bias towards differentiated and class-switched B-cell infiltrates in tumours. Consistent associations were found between B-cell density in tumours, particularly the abundance of memory B cells and more favourable survival outcomes. Despite tumour and immune response heterogeneity, collectively, enriched B-cell signatures such as B-cell abundance, B-cell receptor diversity and immunoglobulin gene rearrangement in tumours correlated with better ICI response. Antibody dysregulation favouring the anti-inflammatory IgG4 isotype was associated with less-favourable outcomes, while class switching to immune-stimulating isotypes such as IgG1 correlated with better clinical outcomes and ICI response. Antibody reactivity and autoantibody analysis revealed distinct isotype signatures in patients, the presence of cancer antigen-reactive antibodies and an association between increased autoantibody production on treatment with ICIs and the development of toxicity (immune-related adverse events; irAEs).

**Conclusions:**

We draw consensus for associations between class-switched B cells and immune-active antibody isotypes that indicate heightened classical immunity, with improved immunotherapy response. Alternatively activated, regulatory B cells and immune-inert antibody isotypes are associated with immunosuppression and less-favourable clinical outcomes. We reveal aspects of humoral immunity that offer opportunities to identify predictive biomarkers of immunotherapy response and irAEs.

## Teaser

The clinical significance of B cells and the antibodies they express in human melanoma is an emerging topic of basic and translational research and biomarker discovery. In this scoping review we draw consensus on associations between mature and memory B cells and class-switched antibodies with improved survival and with better responses to immunotherapy. The literature points to links between specific B-cell subsets, antibody isotypes and antibody reactivity features in predicting and monitoring response and toxicities associated with immunotherapy.

What is already known about this topic?

- B cells and their expressed antibodies are emerging contributors to immune responses in melanoma, the most aggressive form of skin cancer.
- Immune checkpoint inhibitor therapy has advanced the treatment of melanoma, yet significant challenges remain in achieving responses in advanced disease and predicting and managing toxicities (immune-related adverse events; irAEs).
- Understanding B-cell and antibody humoral responses may provide important biologic and therapeutic insights to help address these challenges.

What does this study add?

- This is the first scoping review to evaluate B-cell and antibody responses in melanoma, reported in accordance with PRISMA-ScR guidelines.
- Distinct B-cell and antibody signatures in patients, particularly an abundance of memory B cells, are associated with improved immunotherapy responses.
- Alternatively activated and immunosuppressive B cells and antibodies, including IgG4 and IgA, indicate immune evasion and are correlated with less-favourable survival.
- Autoantibody features are associated with irAEs during immunotherapy.

Melanoma elicits strong immune responses, often manifesting as tumour-infiltrating immune cells,^1,2^ altered circulating immune cell phenotypes and mediators that participate in disease course and treatment responses.^3–6^ T-cell-focused research identified checkpoint molecules, including programmed cell death protein 1 (PD-1), its ligand PD-L1, cytotoxic T-lymphocyte associated protein 4 (CTLA-4) and lymphocyte activation gene 3 (LAG-3), validated targets for immune checkpoint inhibitor (ICI) therapy, which is now the standard-of-care for advanced melanoma.^7–9^ Furthermore, inhibitors of the mitogen-activated protein kinase pathway [e.g. vemurafenib and dabrafenib (anti-BRAF) and trametinib (anti-MEK)] are approved for treating *BRAF* V600E/V600K mutant melanomas.^10–12^

Alongside T cells, B cells and their expressed antibodies are emerging contributors to systemic and tumour-resident pro- and antitumour responses.^13–18^ T helper 2 (Th2)-biased inflammatory conditions in the tumour microenvironment (TME) support regulatory B cells (Bregs) that release anti-inflammatory cytokines.^19–26^ B cells secrete antibodies that trigger antibody-dependent cell-mediated cytotoxicity and phagocytosis, activate complement by binding cognate Fc receptors and complement component-1,^27,28^ and are integral components of tertiary lymphoid structures (TLS).

The abundance, phenotype and location of B cells and their antibodies in melanoma probably influence progression, prognosis and treatment response. However, a comprehensive, global evaluation of the humoral response to melanoma and its clinical significance is lacking. We undertook a scoping review to provide a detailed and systematic search and evaluation of the literature of phenotypic and functional B-cell and antibody profiles and their prognostic and predictive value in human melanoma.

## Materials and methods

### Data sources and searches

This scoping review was reported in accordance with the PRISMA-ScR (https://www.prisma-statement.org/scoping) reporting guidelines (Table S1; see Supporting Information). Inclusion criteria and search strategies are detailed in the review registration on PROSPERO (CRD42024592965). We performed literature searches using three search engines to maximize the search results (PubMed, Scopus and Ovid MEDLINE), using the search terms presented in Table S2 (see Supporting Information). We included full-text studies published in English between 1 January 2000 and 9 October 2024, due to significant advances in technologies and immunological research (the results of each search are provided in Table S3; see Supporting Information). All studies were imported into Rayaan (https://www.rayyan.ai/) for duplication removal and screening. Double-screening by three reviewers (L.B., K.S., X.C.) was conducted for preliminary title and abstract screening, followed by full-text assessment for final screening and inclusion.

### Study selection

We aimed to identify all relevant published studies on B cells and/or antibody responses in human melanoma. Inclusion criteria considered full-text original research articles, published in English and on adults only. Case studies, reviews, preprints and studies exclusively using animals or cell lines were excluded. Prior to data extraction, each study selected for inclusion underwent critical appraisal according to the Joanna Briggs Institute (JBI) critical appraisal tools (https://jbi.global/critical-appraisal-tools). Each study was assessed for quality using the checklists detailed in Table S4 (see Supporting Information). The results are provided in Tables S5–S7 (see Supporting Information). All studies received moderate-to-high scores on JBI assessment, so no studies were excluded at this stage.

### Data extraction

For each study, the following characteristics were extracted into a designated datasheet: first author name, year of publication, study design, participant numbers and demographics, main findings and conclusions. Summary data for each study are provided in Table S8 (see Supporting Information).

### Data analysis

A narrative synthesis organized by key themes was used to summarize and present the findings of the studies included in this scoping review. We classified studies in four research categories: phenotype and function (*n* = 25); prognosis and survival (*n* = 27); response to treatment (*n* = 18); and antibody function and reactivity (*n* = 10). For studies investigating relevant data across categories, data were extracted and analysed in the appropriate category, ensuring that relevant information was not overlooked.

## Results

### Screening and selection of studies

**Figure 1:**
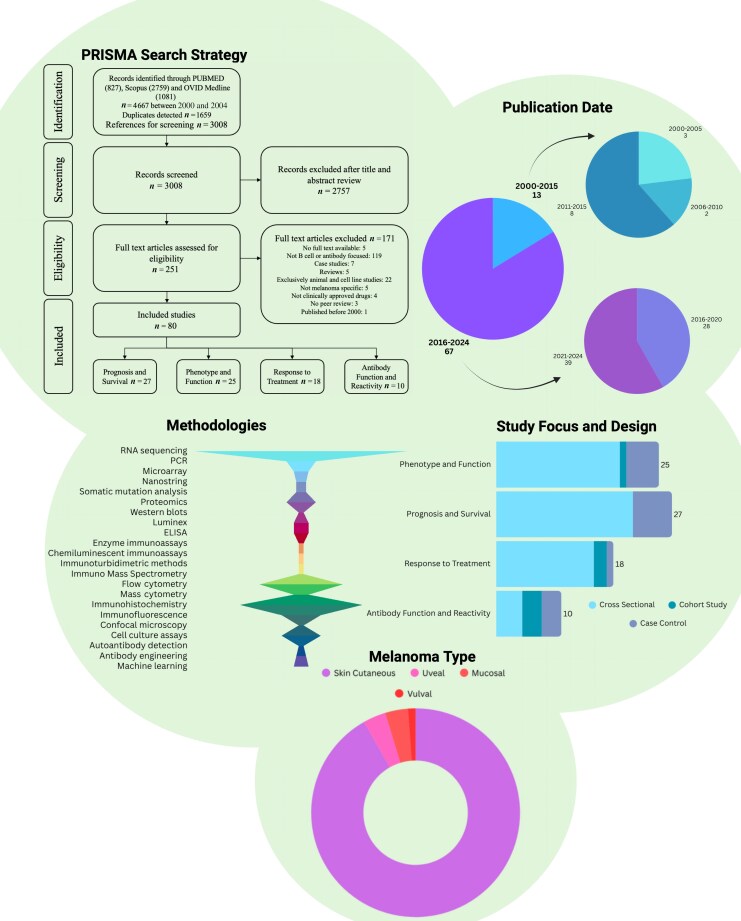
Summary of studies included in the scoping review. ‘PRISMA search strategy’: the PRISMA flow diagram summarizes the results of the search strategy and the subsequent screening process to identify the final 80 included studies. ‘Publication date’: most studies (*n* = 67) were published between 2016 and 2024; only 13 were published between 2000 and 2015. ‘Methodologies’: 22 different techniques and types of assays were used, with the most used highlighted with a wider coloured bar. ‘Study focus and design’: studies were allocated into one of four defined categories based on their broad study focus: phenotype and function (*n* = 25); prognosis and survival (*n* = 27); response to treatment (*n* = 18); and antibody function and reactivity (*n* = 10). The study design was also reported within each category: phenotype and function (case–control, *n* = 5; cohort, *n* = 1; cross-sectional studies, *n* = 19); prognosis and survival (case–control, *n* = 6; cross-sectional studies, *n* = 21); response to treatment (case–control, *n* = 1; cohort, *n* = 2; cross-sectional studies, *n* = 15); and antibody function and reactivity (case–control, *n* = 3; cohort, *n* = 3; cross-sectional studies, *n* = 4). ‘Melanoma type’: Melanoma type was also assessed, with the largest proportion of studies using patient samples from cutaneous (*n* = 78), uveal (*n* = 3), mucosal (*n* = 3) and vulval (*n* = 1) melanoma. ELISA, enzyme-linked immunosorbent assay; PCR, polymerase chain reaction. Figure created with BioRender.com.

We identified 4667 studies through three search engines: PubMed (*n* = 827), Scopus (*n* = 2759) and Ovid MEDLINE (*n* = 1081): Rayaan detected 1659 duplicates, which were removed. After the removal of duplicates, 3008 studies were screened for inclusion based on their titles and abstracts and 251 full-text studies were assessed for inclusion eligibility. This resulted in the inclusion of 80 studies. The study flowchart is presented in Figure 1.

### Phenotype and function of B cells in patients with melanoma

#### B-cell phenotype and frequency in patients

**Figure 2:**
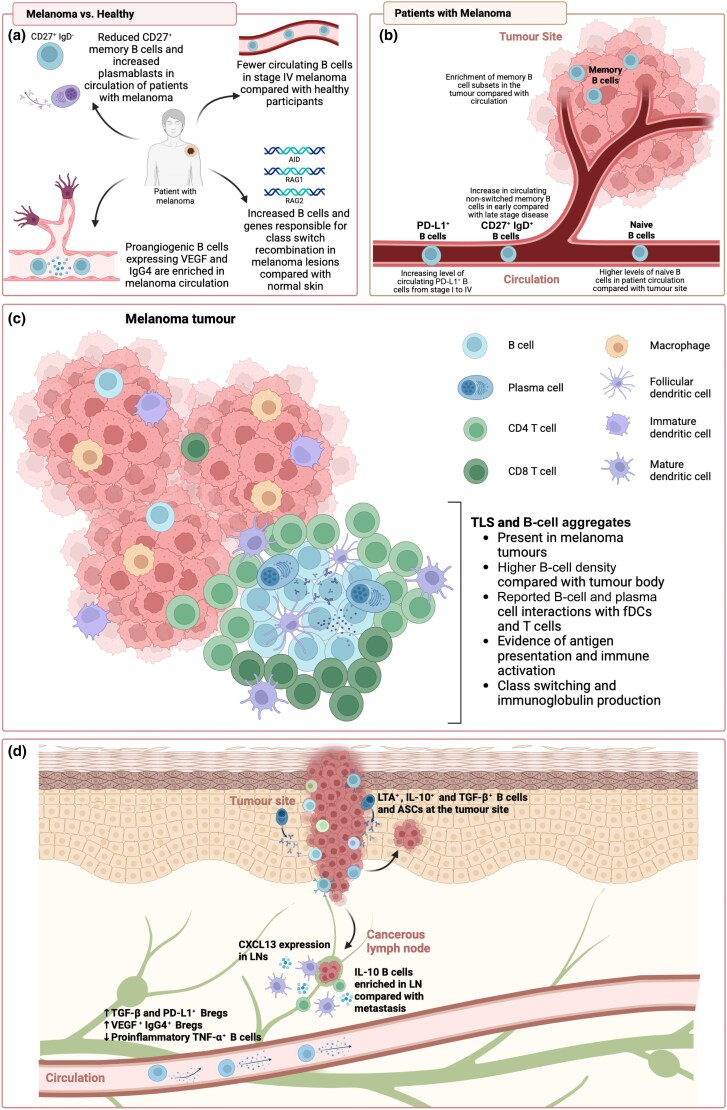
Summary of the findings of studies investigating the phenotype and function of B cells in human melanoma. (a) Studies compared B-cell signatures between groups of patients with melanoma and healthy participants. Studies report reduced CD27^+^ memory B cells and increased plasmablasts in the circulation of patients with melanoma and fewer circulating B cells in those with advanced stage melanoma, and identify proangiogenic B-cell enrichment in melanoma, as well as genes responsible for class-switch recombination. (b) Studies also compare B-cell signatures in melanoma states and report increased naïve, nonswitched memory and programmed death ligand 1 (PD-L1)^+^ B cells in the circulation and enrichment of memory B cells in the tumour. (c) Studies highlight the role of B cells in tertiary lymphoid structures (TLS) and B-cell aggregates. They have been identified in melanoma tumours, where there are high B-cell densities, an abundance of immune cell interactions and antigen presentation, immune activation and class-switching processes. (d) Studies report roles of different cytokine-expressing B cells in melanoma. In the circulation there is an upregulation of transforming growth factor (TGF)-β^+^, PD-L1^+^ and vascular endothelial growth factor (VEGF)^+^ IgG4^+^ regulatory B cells, and a downregulation of tumour necrosis factor (TNF)-α^+^ B cells. CXCL13 expression is reported in lymph nodes (LNs), as well as enrichment of interleukin (IL)-10^+^ B cells compared with metastatic sites. In the tumour, lymphotoxin-alpha (LTA)^+^, IL-10^+^, TGF-β^+^ B cells and antibody-secreting cells are reported. ASC, antibody secreting cells; fDC, follicular dendritic cell. Figure created with BioRender.com.

Seven studies investigated B-cell phenotype or frequency (Table S9; see Supporting Information). Five case–control studies used combinations of flow cytometry, mass cytometry and single-cell RNA sequencing to study circulating B cells in patients and healthy control participants (Figure 2a).^14,29–32^ Studies found lower circulating memory B-cell frequencies,^30,31^ higher levels of circulating plasmablasts,^30^ CD49b^+^ CD73^+^ Bregs^29^ in melanoma and lower overall circulating B-cell levels in patients with stage IV melanoma^32^ compared with healthy participants. Circulating unswitched memory (CD27^+^ IgD^+^) and PD-L1^+^ B cells presenting IgD/IgM naïve phenotypes increased from stage I to IV melanoma (Figure 2b).^32,33^

Three studies reported phenotypic differences between circulating and tumour-resident B cells (Figure 2b).^29,30,34^ Proangiogenic and regulatory [vascular endothelial growth factor (VEGF)^+^ CD73^+^, IgG4^+^ CD73^+^] B cells were identified in tumours.^29^ Lower frequencies of naïve and higher proportions of memory B cells were reported in tumours compared with matched blood.^30^ Furthermore, memory-like B cells (CD19^+^ CD20^+^ CD38^–^ CD138^–^ CD27*^var^*) and plasma cell (PC)-like cells (CD19^+^ CD20^–^ CD138^+^) were enriched at primary tumour sites and distant metastases, respectively.^34^

#### B-cell aggregates and tertiary lymphoid structures in the tumour microenvironment

Six studies used multiplex immunohistochemistry to assess B-cell aggregates and TLS (Figure 2c; Table S9).^35–40^ Three studies identified TLS in the TME,^35–37^ reporting higher TLS density in metastatic vs. primary lesions,^35^ enriched B cells in metastatic melanoma germinal centres and TLS compared with tumour body, and B cells and PCs interacting with follicular dendritic cells and T cells.^36^ The other three studies identified B-cell aggregates in melanoma lesions,^38–40^ surrounded by T cells, dendritic cells and lymphocytes expressing T-cell activation markers.^39^

#### Functional capacity of cytokine and chemokine production by B cells

Five studies investigated cytokine and chemokine expression in melanoma (Figure 2d; Table S9).^14,29,33,41,42^ Four of them explored cytokine expression by circulating and tumour-infiltrating B cells.^14,29,33,41^ Transforming growth factor (TGF)-β^+^ PD-L1^+^ Bregs were enriched, while tumour necrosis factor (TNF)-α^+^ B cells were reduced in patients’ circulation compared with healthy blood.^14^ TGF-β^+^ B cells were also detected in the TME, localized in aggregates with T cells, found across the B-cell differentiation spectrum and not associated with a specific lineage.^14^

Another study found memory-like B cells expressing lymphotoxin alpha (LTA) and interleukin (IL)-10 at the invasive tumour–stroma front, and IL-10^+^ B cells enriched in lymph nodes compared with cutaneous metastases.^41^ VEGF-expressing proangiogenic B cells were identified in tumours and upregulated in patient compared with healthy blood.^29^ Circulating PD-L1^+^ B cells expressed lower levels of regulatory cytokines IL-10, TGF-β and IL-35 compared with total B cells.^33^ Naïve PD-L1^+^ B cells inhibited T-cell-produced interferon-γ in a PD-L1-dependent manner.^33^ Furthermore, the B-cell chemoattractant CXCL13 was the most frequently expressed chemokine in tumours, denoting follicle formation,^42^ and consistent with B–T-cell crosstalk.^14^ Overall, studies consistently report differential B-cell phenotypes in melanoma vs. healthy states, dynamic roles in lymphoid aggregates and TLS, and largely immunosuppressive cytokine-expressing B cells in patient blood and tumours.

### Prognosis and survival

**Figure 3:**
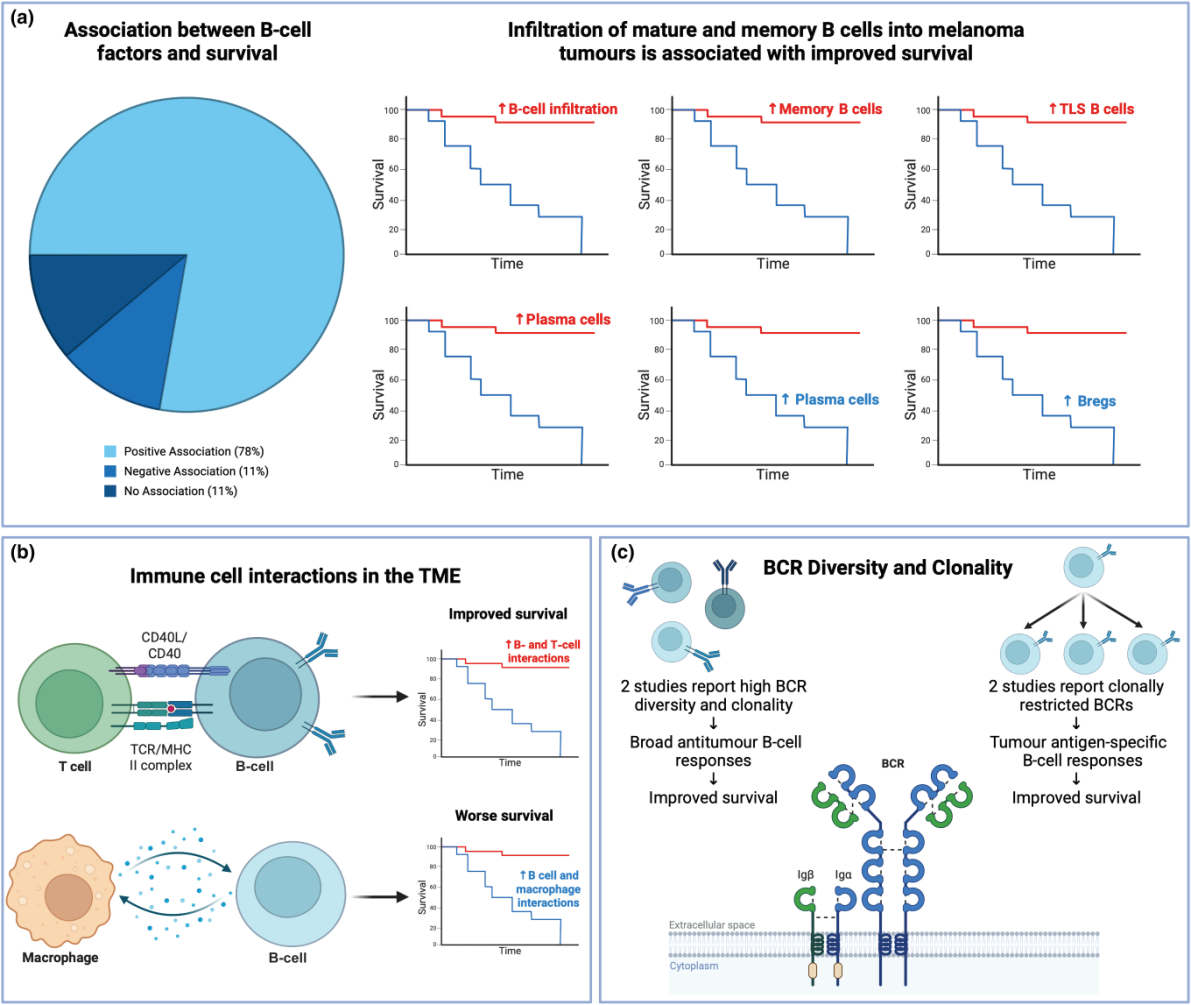
Results of studies reporting on the association between B-cell factors and prognosis and survival. (a) Across all the studies in this category, 78% report a positive association (*n* = 21), 11% a negative association (*n* = 3) and 11% no association (*n* = 3) between B-cell factors and survival. Key B-cell factors associated with improved survival are high B-cell infiltration, memory B cells and tertiary lymphoid structure (TLS)-associated B cells. Regulatory B cells (Bregs) are associated with worse overall survival (OS). Studies show that high levels of plasma cells in the tumour are associated with better survival (e.g. Huang *et al*.),^48^ while others report associations with worse survival (e.g. Bosisio *et al*.).^64^ (b) Studies report that interactions in the tumour microenvironment between B and T cells are usually activatory and are therefore associated with improved OS; however, B-cell and macrophage interactions have been reported as immunosuppressive and are therefore correlated with worse prognosis. (c) There are conflicting reports on B-cell receptor (BCR) diversity and survival. Two studies report that high BCR diversity and clonality denote broad antitumour responses,^44,55^ resulting in improved survival, and two studies report that clonally restricted BCRs denote tumour antigen-specific B-cell responses and are therefore also associated with improved survival.^49,56^ MHC, major histocompatibility complex; TCR, T-cell receptor. Figure created with BioRender.com.

Of 27 studies [Table 1; Table S10 (see Supporting Information)], 21 reported positive,^43–63^ 3 reported negative^64–66^ and 3 reported no associations^67–69^ between B-cell-related factors and patient survival pretreatment (Figure 3a).

**Table 1:** Studies investigating the association between B-cell responses in melanoma and survival outcomes

Study	Cohort details	Tumour stage	Association between B cells and survival
Attrill 2022^43^	Primary melanoma (*n* = 66)	Primary (stage I/II) and metastatic (stage III)	Positive
	• Stage II (*n* = 39)		
	• Stage III (*n* = 27)		
Cabrita 2020^44^	Patients with melanoma (*n* = 177)	Primary (stage II) and metastatic (stage III/IV)	Positive
	• Stage II (*n* = 19)		
	• Stage III (*n* = 104)		
	• Stage IV (*n* = 50)		
	• Unknown (*n* = 4)		
	The study also included smaller cohorts of patients treated with anti-CTLA-4 (*n* = 37) and anti-PD-1 (*n* = 40)		
Freeman 2022^45^	Primary cohort: patients with melanoma with WES (*n* = 189) and RNAseq (*n* = 178) data	Primary (stage II) and metastatic (stage III/IV)	Positive
	Secondary cohort: patients with melanoma treated with anti-PD-1 or combination anti-CTLA-4/PD-1 (*n* = 180)		
García-Mulero 2021^46^	Patients with primary uveal melanoma (*n* = 213) from 5 datasets (TCGA and GEO repositories)	Primary (stage I/II)	Positive
Garg 2016^47^	Cohort 1: primary cutaneous melanoma (*n* = 57)	Primary (stage II) and metastatic (stage III/IV)	Positive
	• No metastasis (*n* = 43)		
	• Metastasis at diagnosis (*n* = 14)		
	Cohort 2: primary cutaneous melanoma (*n* = 41)		
	• No metastasis (*n* = 25)		
	• Metastasis at diagnosis (*n* = 16)		
	Cohort 3: cutaneous melanoma samples from TCGA (*n* = 345)		
Huang 2023^48^	Patients with melanoma patients (*n* = 467) from TCGA (*n* = 233 with *BRAF* mutations, 50%)	Primary (stage II) and metastatic (stage III/IV)	Positive
Iglesia 2016^49^	Samples from patients with melanoma (*n* = 329)	Primary (stage II) and metastatic (stage III/IV)	Positive
Kang 2020^50^	Patients with cutaneous melanoma (*n* = 449) from TCGA	Primary (stage II) and metastatic (stage III/IV)	Positive
	• Stage 0/I (*n* = 91)		
	• Stage II (*n* = 133)		
	• Stage III (*n* = 168)		
	• Stage IV (*n* = 22)		
	• Unknown (*n* = 35)		
Lardone 2016^51^	Datasets included patients with stage III and IV metastatic melanoma from publicly available studies: GSE22153 (*n* = 57 patients), GSE46517 (*n* = 25 primary cutaneous and *n* = 61 metastatic melanoma) and GSE19234 (*n* = 44 metastatic melanoma tissue samples from 38 patients)	Metastatic (stage III/IV)	Positive
Lundberg 2022^52^	Patients with melanoma patients (*n* = 325)	Primary (stage II) and metastatic (stage III)	Positive
	• Anti-PD-1-treated (*n* = 121) and anti-CTLA-4-treated (*n* = 40) melanoma patients for ICI response		
Lynch 2021^53^	Patients with stage IIIB–IV cutaneous melanoma metastases (*n* = 64)	Metastatic (stage III/IV)	Positive
Martínez-Escribano 2003^54^	Patients with primary cutaneous melanoma (*n* = 38) and healthy control participants (*n* = 27)	Primary (stage I/II)	Positive
Schina 2023^55^	Data sourced from TCGA, 30 cancer types and 10 additional immunotherapy datasets; melanoma data from patients with advanced melanoma treated with anti-PD-1 therapy (*n* = 268)	Metastatic (stage III/IV)	Positive
Selitsky 2019^56^	Patients with cutaneous melanoma from TCGA (*n* = 473)	Primary (stage II) and metastatic (stage III)	Positive
Therien 2022^57^	Flow cytometry cohort: patients with melanoma (*n* = 13; 3 with tumour in SLN, 10 without)	Primary (stage I/II) and metastatic lymph node (III)	Positive
	DSP cohort: patients with melanoma (*n* = 24; 8 with tumour in SLN, 16 without)		
Versluis 2024^58^	Patients with stage III melanoma (*n* = 98), (49 observation, 49 adjuvant intention)	Metastatic (stage III)	Positive
Wang 2022^59^	A total number of 4645 cells from patients with melanoma (*n* = 19)	Primary (stage II) and metastatic (stage III/IV)	Positive
Xiong 2020^60^	Patients with melanoma [*n* = 469: stage 0/I/II (42.5%), stage III/IV (46.6%)]: LN tissue (*n* = 221), *in situ* SKMC tissue (*n* = 103), distant metastatic tissue (*n* = 68), adjacent tissue (*n* = 74) and unidentified tissue sources (*n* = 3)	Primary (stage II) and metastatic (stage III/IV)	Positive
Yan 2020^61^	Patients with SKCM from TCGA (*n* = 454)	Primary (stage II) and metastatic (stage III/IV)	Positive
	• Stage I/II (49.8%)		
	• Stage III/IV (42.3%)		
	• Unknown stage (7.9%)		
Zhao 2022^62^	Primary cohort: patients with uveal melanoma from TCGA (*n* = 80)	Primary (stage II) and metastatic (stage III/IV)	Positive
	Validation cohorts: patients with uveal melanoma from GSE22138 (*n* = 63) and patients with uveal melanoma from GSE84976 (*n* = 28)		
Zhou 2024^63^	Healthy skin tissue samples (*n* = 812; GTEx database); patients with melanoma (*n* = 451; TCGA dataset). Patients analysed for gene mutations (*n* = 469)	Primary (stage II) and metastatic (stage III/IV)	Positive
Andrés 2006^67^	Patients with melanoma (*n* = 86): disease-free (*n* = 63) and with distant metastases (*n* = 23)	Primary (stage II) and metastatic (stage III/IV)	None (no survival analyses conducted)
Damsky 2019^68^	Patients with melanoma treated with anti-PD-1 therapy (*n* = 40)	Primary (stage II) and metastatic (stage III/IV)	None
Hillen 2008^69^	Patients with melanoma (*n* = 58); superficial spreading melanoma (*n* = 37), 36% nodular melanoma (*n* = 21)	Primary (stage II) and metastatic (stage III/IV)	None
Bosisio 2016^64^	Primary cutaneous melanomas (*n* = 710)	Primary (stage I/II)	Negative
Brase 2021^65^	Patients with melanoma (*n* = 146); all had *BRAF* V600-mutant metastatic melanoma	Metastatic (stage III/IV)	Negative
	*BRAF* mutation: V600E (91%), V600K (8%)		
	Metastasis stage: M0 (5%), M1 (95%)		
Martinez-Rodriguez 2014^66^	Primary cutaneous melanoma (*n* = 91): 51 women and 40 men aged 21–87 years	Primary (stage I/II)	Negative

CTLA-4, cytotoxic T-lymphocyte associated protein 4; DSP, digital spatial profiling; GEO, Gene Expression Omnibus; ICI, immune checkpoint inhibitor; LN, lymph node; PD-1, programmed cell death protein 1; RNAseq, RNA sequencing; SKCM, skin cutaneous melanoma; SLN, sentinel lymph node; TCGA, The Cancer Genome Atlas; WES, whole-exome sequencing.

#### Tumour-infiltrating B cells are associated with prognosis in patients with melanoma

Of 18 studies,^43,44,46–48,50–52,54,56–59,61,62,64–66^ 15 reported higher B-cell infiltration (especially memory B cells, PCs or TLS-resident B cells) associated with improved prognosis and survival (Figure 3a).^43,44,46–48,50–52,54,56–59,61,62^ One study found enrichment of memory B cells linked to improved survival, while Breg signatures were associated with worse prognosis (Figure 3a).^56^

Three studies found worse outcomes in patients with high B-cell and PC infiltration (Figure 3a):^64–66^ high proportions of tumour-infiltrating B cells were associated with worse prognosis,^65,66^ and tumour-infiltrating PC clusters were correlated with worse survival.^64^ In patients with *BRAF*-mutant melanoma, high baseline B-cell gene signatures were linked to reduced overall survival (OS) following BRAF/MEK inhibitor therapy.^65^

#### B-cell interactions with other immune cells

Five studies investigated B-cell interactions with other immune cells in the TME (Figure 3b).^44,48,51,57,70^ Three studies reported B–T-cell interactions resulting in immune activation associated with improved survival.^44,51,57^ Two reported immunosuppressive macrophages alongside B cells corresponding with worse prognosis.^48,70^

#### B-cell receptor diversity and clonality correlate with survival outcomes

Four studies assessed whether B-cell receptor (BCR) diversity and clonality affect prognosis (Figure 3c).^45,49,55,56^ Two studies reported high BCR diversity and clonality, denoting broad antigen-driven responses, linked to favourable OS.^45,55^ The other two papers found that restricted BCR diversity was associated with improved survival outcomes:^49,56^ low BCR diversity was associated with improved survival and clonally restricted BCR signatures as favourably prognostic.^49,56^

Overall, studies of patients pretreatment reported associations between mature and differentiated B-cell phenotypes with more favourable outcomes; few studies reporting poorer prognosis found associations with PC infiltrates and immunosuppressive/regulatory tumour-infiltrating B cells. Survival outcomes may be partly dependent on intratumoral B-cell interactions with surrounding immune or cancer cells. Conflicting findings relate to BCR signatures and survival outcomes, implicating antigenic specificity in prognosis.

### Treatment response

**Figure 4:**
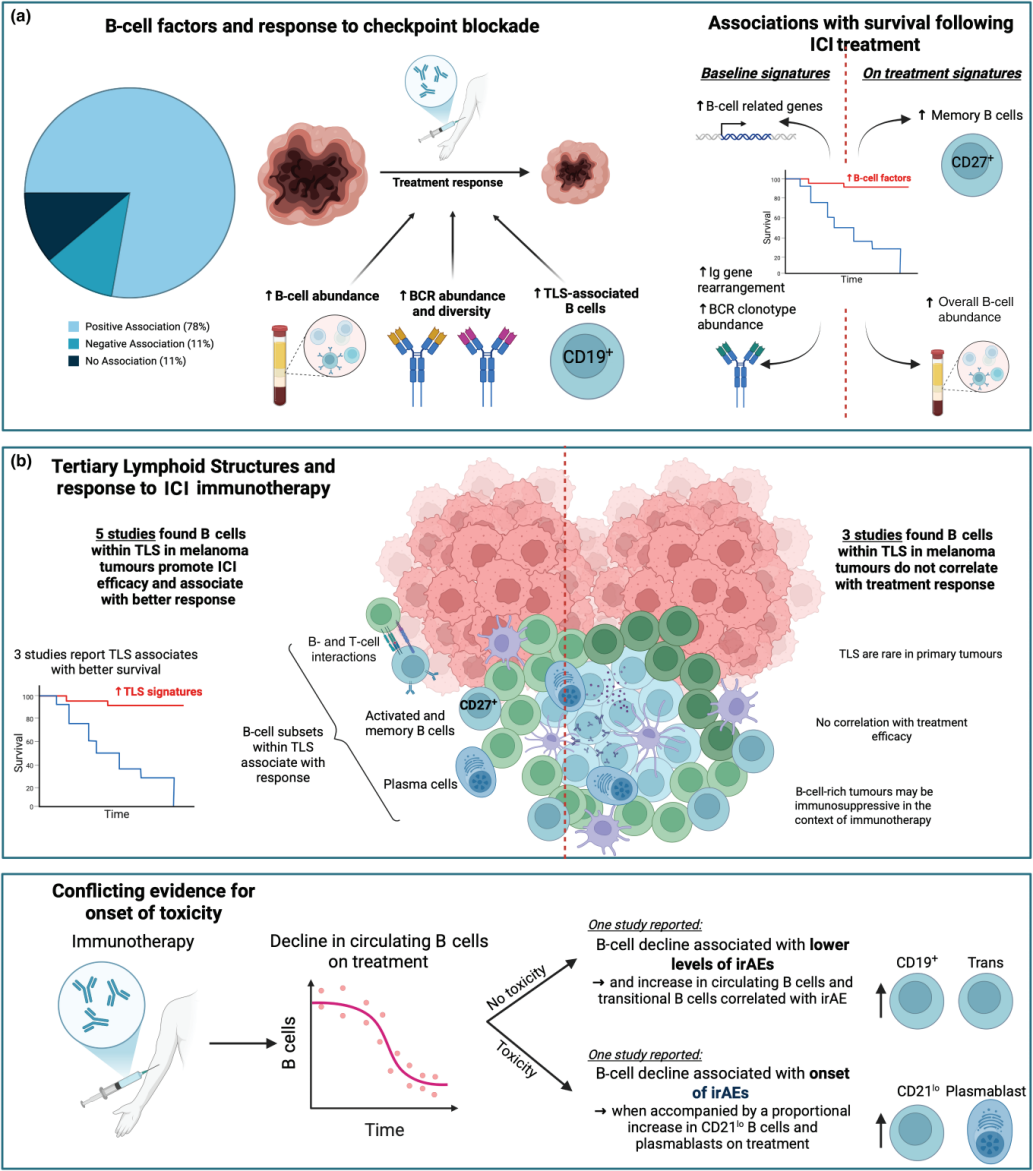
Results of studies reporting on the association between B-cell factors and response to treatment. (a) The pie chart shows the number of studies correlating B-cell responses with response to treatment: positive association (78%; *n* = 14), no association (11%; *n* = 2) and negative association (11%; *n* = 2). Three key B-cell factors are found to be associated with improved responses to immune checkpoint inhibitor (ICI) therapy, including increased B-cell abundance, B-cell receptor (BCR) abundance, and diversity and presence of tertiary lymphoid structure (TLS)-associated B cells. In patients with improved survival after ICI treatment, studies report enriched B-cell-related genes and increased immunoglobulin (Ig) gene rearrangement and BCR clonotype abundance at baseline, and increased memory B cells and overall B-cell abundance on treatment. (b) Studies report an association between the presence of TLS and response to ICI therapy. Five studies report that B cells in TLS promote ICI efficacy and are associated with better response,^44,53,75,79,82^ highlighting the proinflammatory B- and T-cell interactions, an abundance of activated, memory B cells and plasma cells, and found that TLS is associated with better survival in these patients. Three studies report that B cells in TLS are not correlated with treatment response and that TLS are rare in primary tumours;^64,65,68^ they also report the immunosuppressive properties of B-cell-rich tumours in the context of immunotherapy. (c) Two studies report a decline in circulating B cells after ICI treatment but present conflicting results in how this decline is correlated with the onset of immune-related adverse events (irAEs).^72,84^ Despite an overall decline in B cells after treatment, both studies report proportional increases in specific B-cell subsets while on treatment that correlated with irAE development. ICI, immune checkpoint inhibitor. Figure created with BioRender.com.

Eighteen studies [Table 2; Table S11 (see Supporting Information)] investigated B-cell signature and frequency and treatment response: 14 reported positive associations,^70–83^ 2 negative associations^84,85^ and 2 no association (Figure 4a).^86,87^

**Table 2:** Studies investigating the association between B-cell signatures in melanoma and response to treatment

Study	Cohort details	Tumour stage	Therapeutic agent	Association between B cells and response to treatment
Anagnostou 2020^71^	Patients with melanoma treated with anti-PD-1, anti-CTLA-4 or a combination (*n* = 64)	Metastatic (stage III/IV)	Anti-PD-1 and anti-CTLA-4 ICI therapy	Positive
Das 2018^72^	Patients with advanced melanoma receiving ICI therapy, including combination (*n* = 23), anti-CTLA-4 (*n* = 8) and anti-PD-1 (*n* = 8) monotherapy	Metastatic (stage IV)	Anti-PD-1 and anti-CTLA-4 ICI therapy	Positive
Ding 2023^73^	Tumour samples from patients with melanoma (*n* = 48) treated with ICI therapy	Metastatic (stage III/IV)	Anti-PD-1 and anti-CTLA-4 ICI therapy	Positive
Dollinger 2020^70^	Metastatic melanoma (*n* = 32), treated with anti-PD-1, anti-CTLA-4 or combination therapy	Metastatic (stage III/IV)	Anti-PD-1 and anti-CTLA-4 ICI therapy	Positive
Egan 2023^74^	Discovery dataset: patients with metastatic melanoma (*n* = 19) treated with ICI therapy	Metastatic (stage III/IV)	Anti-PD-1 and anti-CTLA-4 ICI therapy	Positive
	Validation dataset: patients from four independent studies (*n* = 209)			
	Patients with advanced melanoma treated with anti-PD-1, anti-CTLA-4 or combination			
Helmink 2020^75^	Patients with melanoma on neoadjuvant ICI therapy (*n* = 23), with nivolumab (*n* = 12) or ipilimumab (*n* = 11)	Metastatic (stage III)	Anti-PD-1 and anti-CTLA-4 ICI therapy; targeted BRAF and MEK inhibitors	Positive
	Validation cohort included patients with stage III melanoma enrolled in OpACIN-neo trial (*n* = 18)^a^			
	Targeted therapy cohort of patients who received DAB-TRAM for *BRAF*-mutated melanoma (*n* = 13)			
Liu 2021^76^	TCGA Skin Cutaneous Melanoma dataset (*n* = 472), GSE65904 (*n* = 214), GSE98394 (*n* = 78), GSE53118 (*n* = 79), ICI melanoma samples from RNAseq datasets (*n* = 182), ICI melanoma samples from microarray dataset (*n* = 65), GSE720564 (485 single cells)	Primary (stage II) and metastatic (stage III/IV)	Anti-PD-1 and anti-CTLA-4 ICI therapy	Positive
Onieva 2022^77^	Discovery cohort of patients with metastatic melanoma treated with nivolumab (*n* = 21)	Metastatic (stage III/IV)	Anti-PD-1 immunotherapy	Positive
	Validation cohort of patients with melanoma treated with anti-PD-1 (*n* = 32)			
Pourmaleki 2022^78^	Patients with in-transit melanoma metastases (initial cohort *n* = 7, validation cohort *n* = 19); patients treated with either intralesional or high-dose systemic IL-2	Metastatic (stage III/IV)	IL-2 therapy	Positive
Quek 2024^79^	Patients with metastatic melanoma (*n* = 5); patients were treated with anti-PD-1 (nivolumab or pembrolizumab) and/or anti-CTLA-4 (ipilimumab) therapy	Metastatic (stage III/IV)	Anti-PD-1 and anti-CTLA-4 ICI therapy	Positive
Valpione 2022^80^	Validation cohort of pretreatment melanoma biopsies (*n* = 20) and pretreatment (anti-PD-1) melanoma samples from published datasets (*n* = 120)	Metastatic (stage IV)	Anti-PD-1 ICI therapy	Positive
Varn 2019^81^	Patients with melanoma receiving anti-PD-1 inhibitors (*n* = 28) and anti-CTLA-4 inhibitors (*n* = 42)	Metastatic (stage III/IV)	Anti-PD-1 and anti-CTLA-4 ICI therapy	Positive
Wu 2022^82^	Patients with melanoma treated with anti-PD-1 therapy split into those whose disease responded to treatment (*n* = 17) and those whose disease did not (*n* = 31)	Metastatic (stage III/IV)	Anti-PD-1 ICI therapy	Positive
Zhang 2024^83^	79 patients included in the single-cell dataset; 6 patients receiving anti-PD-1 or anti-CTLA-4 were included in the bulk dataset	Metastatic (stage III/IV)	Anti-PD-1 and anti-CTLA-4 ICI therapy	Positive
Aklilu 2004^86^	Patients with metastatic melanoma (*n* = 6)	Metastatic (stage III/IV)	Rituximab anti-CD20 and IL-2 therapy	None
Mastracci 2020^87^	Patients with metastatic melanoma (*n* = 17, 11 men, 6 women); median age 62 years; 88% had cutaneous melanoma, 6% had mucosal melanoma	Metastatic (stage III/IV)	Anti-CTLA-4 ICI therapy	None
Gatto 2023^84^	Patients with metastatic melanoma treated with immune checkpoint inhibitors with no irAEs (*n* = 15) and IA (*n* = 7)	Metastatic (stage III/IV)	Anti-PD-1, anti-CTLA-4 ICI and anti-LAG-3 therapy	Negative
Somasundaram 2017^85^	First cohort: therapy-resistant tumour samples from pretreatment and BRAF inhibitor monotherapy or BRAF/MEK inhibitor combination (*n* = 20)	Metastatic (stage III/IV)	Targeted BRAF and MEK inhibitors; CD20 depletion	Negative
	Second cohort: therapy-resistant tumour samples from pretreatment and on treatment with dabrafenib (*n* = 21)			
	Gene expression data (*n* = 52) and RNAseq data (*n* = 38) from metastatic pretreatment and treatment-resistant melanoma tumours			
	Patients with advanced metastatic, therapy-resistant melanoma (*n* = 10), pilot trial of CD20 depletion with ofatumumab – most patients had received multiple previous systemic therapies (*n* = 7)			

CTLA-4, cytotoxic T-lymphocyte associated protein 4; DAB-TRAM, dabrafenib and trametinib; IA, inflammatory arthritis; ICI, immune checkpoint inhibitor; IL, interleukin; irAE, immune-related adverse event; LAG-3, lymphocyte activation gene 3; PD-1, programmed cell death protein 1; RNAseq, RNA sequencing; TCGA, The Cancer Genome Atlas. ^a^NCT02437279.

#### Clinical responses to immunotherapy

Twelve studies investigated associations between B cells and immunotherapy outcomes;^52,58,68,70,71,73–78,87^ eight broadly studied immunotherapy response (Table S11);^70,71,73,75,76–78,87^ four focusing on prognosis and survival also reported associations with response (Table S10).^52,58,68,75^

Eight of these studies reported baseline intratumour signatures such as higher B-cell abundance,^52,70,74,75,77^ increased BCR diversity,^76,77^ immunoglobulin gene rearrangement^71^ and TLS presence^75^ correlated with better ICI response (Figure 4a). Another found that patients whose melanoma responded to ICI therapy had higher B-cell abundance on treatment than those whose melanoma did not.^73^ Three studies reported low B-cell tumour infiltration prior to treatment not linked with anti-PD-1 efficacy;^68^ baseline B-cell scores not predicting response to adjuvant anti-PD-1 in stage III melanoma;^58^ and no differences in B-cell tumour density at baseline between patients whose melanoma responded to anti-CTLA-4 and those whose melanoma did not.^87^

#### Patient survival after immune checkpoint inhibitor treatment

Six studies evaluated associations between B-cell abundance and survival. Increased intratumoral B cells, BCR abundance and immunoglobulin gene signatures were associated with improved survival after ICI treatment.^71,73,77,80,81,83^ Of these, three used pretreatment samples to retrospectively predict response. Baseline upregulation of B-cell-related genes,^77^ immunoglobulin gene rearrangement^71^ and BCR clonotype abundance^80^ were associated with improved progression-free (PFS) survival and OS after ICI treatment. Three studies found higher memory B-cell scores and increased overall B-cell abundance on treatment to be associated with better prognosis in patients treated with ICIs (Figure 4a).^73,83^

#### Association between tertiary lymphoid structure and immunotherapy response

Nine studies reported associations between TLS and ICI response (Figure 4b).^44,53,64,65,68,70,75,79,82^ Of these, five reported that B cells in TLS promote antitumour immunity, synergizing with T cells,^44,53,75,79,82^ three of which correlated TLS formation with PFS, OS and ICI response.^44,75,79^ Three studies identified clonally expanded and functionally activated memory B cells and PCs in tumours that responded to ICI treatment;^75^ a memory B-cell subset (CD20^+^ CD22^+^ ADAM28^+^) that promoted antitumour immunity through myeloid cell interactions;^82^ activation-induced cytidine deaminase (AID)^+^ B cells and activated CD21^+^ B cells correlated with more favourable OS.^53^ Another study reported memory B-cell enrichment in patients whose tumours responded to ICI treatment.^70^ However, three studies reported no correlation between B-cell infiltration with treatment efficacy,^68^ that TLS are rare in primary tumours^64^ and that B-cell-rich tumours may be immunosuppressive in the ICI context.^65^

#### Immune-related adverse events during immune checkpoint inhibitor therapy

Two studies correlated circulating B-cell levels in patients while they were receiving ICI therapy with the onset of immune-related adverse events (irAEs; Figure 4c).^72,84^ A cross-sectional study reported a decline in circulating B cells during treatment in patients who did not experience irAEs, while enriched circulating transitional B cells and circulating CD19^+^ B cells on treatment were reported in patients who developed inflammatory arthritis.^84^ However, an overall circulating B-cell decline and proportional increase in CD21^lo^ B cells and plasmablasts after therapy correlated with irAE onset and worse OS in patients with high-grade toxicity.^72^

#### B-cell responses to different immune checkpoint inhibitor combinations

Four studies compared B-cell signatures between ICI treatments (Table S12; see Supporting Information).^72,79,81,84^ High B-cell gene expression predicted improved PFS and OS with anti-PD-1 monotherapy but not combined anti-PD-1/anti-CTLA-4.^79^ On treatment B-cell decline was correlated with the onset of several toxicities and worse OS in combined anti-PD-1/anti-CTLA-4 compared with monotherapy,^72^ but B-cell frequency did not differ between combination and monotherapy in patients with an irAE of inflammatory arthritis.^84^ Memory B-cell-like scores correlated with improved survival for anti-PD-1 and anti-CTLA-4 monotherapy, while BCR heavy chain abundance was only prognostic in patients on anti-CTLA-4.^81^

#### B cells and clinical responses to other therapies

Four studies assessed B-cell infiltrates in non-ICI therapies.^73,78,85,86^ Rituximab-mediated B-cell depletion showed antitumour activity in BRAF inhibitor-resistant melanoma.^85^ Higher stromal B-cell densities and aggregates were found at baseline in matched untreated and IL-2-injected tumours of patients whose melanoma responded highly to treatment.^78^ However, in another study, depletion of B cells with rituximab did not significantly improve clinical outcomes when combined with IL-2 therapy.^86^ Increased B-cell abundance, TLS signatures and BCR diversity were associated with improved survival after ICI but not BRAF/MEK inhibition,^73^ indicating therapy-specific roles of B cells.

Together, enriched B-cell signatures at baseline and on treatment are linked in patients whose melanoma responds to, and are associated with improved survival after, ICI treatment. Memory B cells in tumours and TLS are associated with ICI response. Few studies have reported on the immunosuppressive roles of B cells in tumours resulting in worse therapy response, highlighting potential dysregulation of B-cell activation in immune checkpoint inhibition and that the proportional enrichment of some B-cell populations on treatment is associated with the onset of irAEs.

### Antibody function and reactivity

#### Immunoglobulin expression in patients

**Figure 5:**
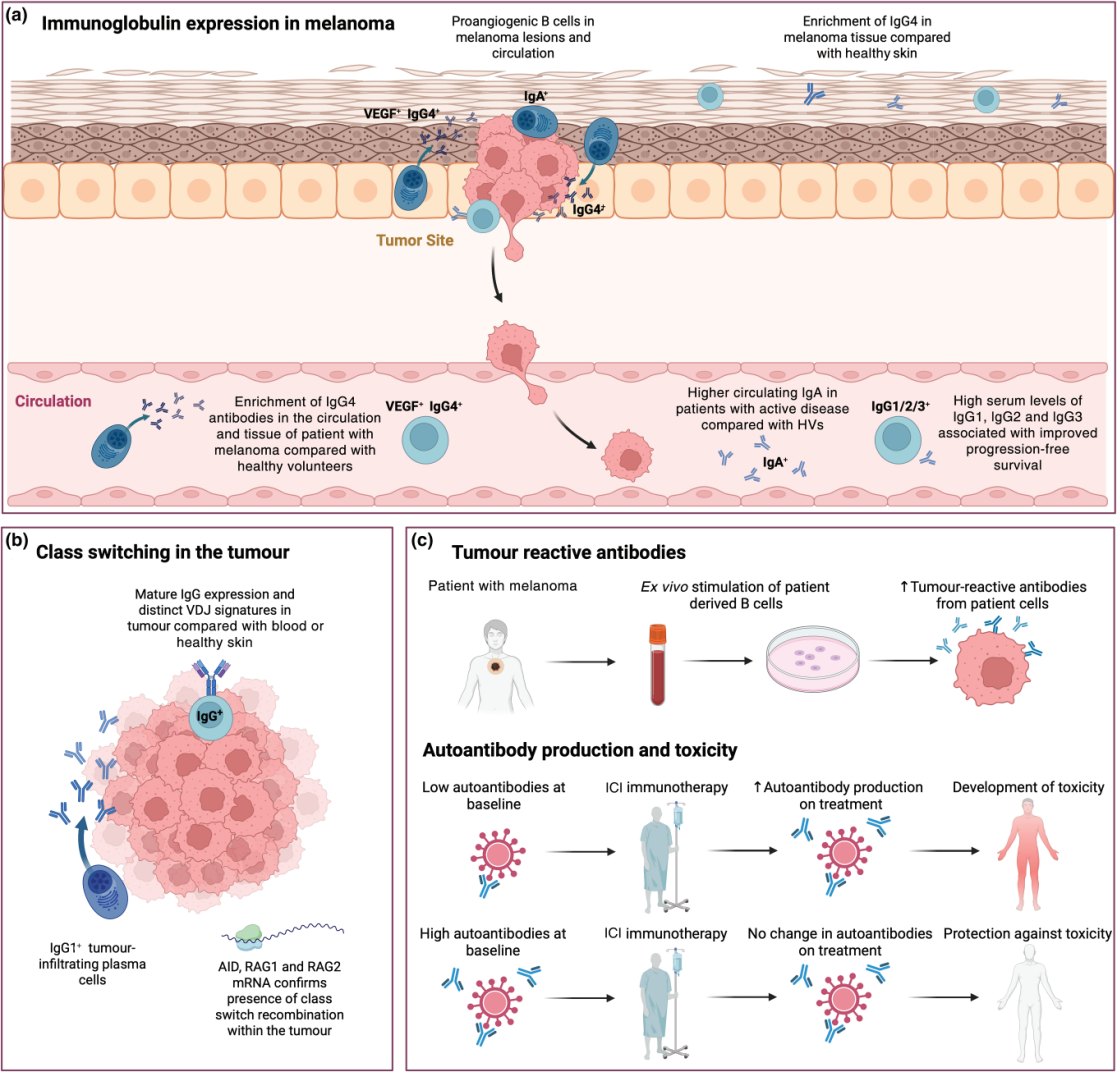
Results of studies reporting on antibody function and reactivity in melanoma patients. (a) Several studies assessed immunoglobulin expression in melanoma. Studies report an enrichment of circulating IgG4 and IgA in patients compared with healthy volunteers (HVs). In patients, high serum levels of IgG1, IgG2 and IgG3 are associated with improved progression-free survival, while high IgG4 is associated with worse prognosis. Class-switched and proangiogenic B cells are identified in the tumour, and enrichment of IgG4 is found in melanoma tissue compared with healthy skin. (b) When assessing class-switching in the tumour, studies report distinct VDJ signatures between the tumour and blood, identify IgG1^+^ plasma cells in the tumour and detect the expression of activation-induced cytidine deaminase (AID), recombination-activating gene (RAG)-1/RAG-2 in the tumour, confirming class-switching in these regions. (c) Tumour reactive antibodies: two studies investigated tumour cell-reactive antibodies from the *ex vivo* grown circulating B cells of patients with melanoma.^15,96^ One study reports that antibodies from patients had higher reactivity to melanoma than antibodies from healthy participants. Autoantibody production and toxicity: studies also correlate the production of circulating autoantibodies with the onset of immune-related adverse events, finding an increase on treatment to be associated with development of toxicity, while high baseline levels and no change on treatment is suggested to be protective against toxicity. ICI, immune checkpoint inhibitor; VEGF, vascular endothelial growth factor. Figure created with BioRender.com.

Seven studies investigated IgG antibodies in melanoma [Figure 5a; Table S13 (see Supporting Information)].^29,67,88–92^ Four case–control studies found increased proportional IgG4 expression by tumour-infiltrating B cells and proportionally higher serum IgG4 in relation to total IgG in patients compared with healthy participants,^29,88–90^ associated with reduced OS.^88,89^ One study found that a significant proportion of IgGs were IgG4 (12%) in tumours, while IgG4 was rare in healthy skin.^90^ Another study reported improved PFS in patients with high serum levels of total IgG, specifically IgG1, IgG2 and IgG3.^91^

One study identified high IgA^+^ PC tumour infiltration,^64^ and another higher circulating IgA levels in patients with active disease compared with healthy participants.^67^ TLS-resident B cells showed clonal amplification, somatic mutation and isotype-switching to IgA, supporting local antigen-driven responses.^93^ Furthermore, high proportional intratumoral IgA and IgG expression were associated with lower and higher survival probability, respectively.^30^

#### Antibody-secreting cells and class-switching mechanisms in tumours

Four studies assessing class-switching processes and PCs detected active class-switching in tumours (Figure 5b; Table S13).^30,90,93,94^ Three studies reported mRNA for the transiently expressed enzyme AID, which is involved in active class-switch recombination and somatic hypermutation, mature IgG transcript expression and distinct VDJ sequence signatures in tumours compared with blood or healthy skin.^30,90,93^ One study reported that tumour-infiltrating PCs predominantly expressed IgG.^94^ While melanoma had the lowest clone distribution among other cancers, it featured high levels of clonal expansion, suggesting that few clones dominate humoral repertoires.^95^

#### Autoantibody production

Two studies reported reactivity of patient circulating B-cell-derived antibodies to melanoma cells,^15,96^ with higher antibody reactivity from patient vs. healthy individual B cells, and reduced antibody responses to melanoma with advanced stage.^15^ Three studies investigated autoantibodies in patients and subsequent response to ICI therapy.^84,97,98^ One study found associations between antithyroid autoantibody development during anti-CTLA-4 treatment and the onset of thyroid dysfunction on subsequent anti-PD-1 therapy.^97^ The other two found increased autoantibodies during ICI therapy associated with irAEs (Figure 5c).^84,98^ Another study found significantly higher serum autoantibody levels against nine antigens, predominantly tubulins and autoimmunity-related proteins, in active disease vs. resected disease and healthy states.^30^

Collectively, IgG4 and IgA isotypes are associated with worse survival. Autoantibody induction on treatment may correlate with specific irAEs (Table S13).

## Discussion

Multiple studies indicate a collapse of memory and enrichment of naïve and regulatory circulating B-cell populations in patients with melanoma compared with healthy study participants.^14,29–32^ An increased frequency of memory phenotypes is consistently reported in tumours compared with peripheral blood.^30,34^ Therefore, while the circulating humoral compartment is enriched in naïve B cells, mature and class-switched B-cell populations are recruited into or differentiate in the TME.^30,99^ B-cell aggregates and TLS in tumours are widely reported,^35–40^ denoting roles in lymphoid assembly and attempts to stimulate adaptive immune responses *in situ*. Circulating and tumour-infiltrating B cells express immunosuppressive factors/cytokines (e.g. TGF-β, IL-10, LTA, PD-L1 and VEGF)^14,29,41^ that may promote impaired antitumour responses. Together, these denote mature, yet alternatively activated and immunosuppressive, B-cell signatures in melanoma.

Most studies have reported positive prognostic values of B cells for survival. Infiltration of memory B cells and TLS-associated B cells in tumours is correlated with improved prognosis.^43,44,46–48,50–52,54,56–59,61,62,82^ In contrast, infiltrating PC sheets and immunosuppressive B-cell features are associated with worse prognosis,^56,64,65^ probably representing roles in pathogenesis or bystander effects of increased inflammation alongside aggressive disease. PC sheets found in thicker, ulcerated and mitotically active tumours with rare TLS formation may denote extrafollicular differentiation without lymphoid assembly and T-cell–B-cell interaction,^64^ explaining the opposing findings that higher PC frequencies are associated with better prognosis.^48^ In *BRAF*-mutant tumours of patients who had better outcomes, PCs were detected alongside naïve B cells and CD8^+^/CD4^+^ memory/activated T cells, forming immune-rich conditions conducive to cross-talk and antigen presentation *in situ*.^48^ Future research should assess cohort-specific prognostic (e.g. pathway-dysregulated/*BRAF*-mutant melanoma) and different predictive values for B cells and PCs in targeted vs. ICI treatments.

Two studies exclusively-focused on uveal melanoma.^46,62^ Similarly to cutaneous melanoma, B-cell tumour infiltration was associated with better prognosis.^46^ A protective role of naïve B cells was suggested, where low tumour infiltration was correlated with worse survival.^62^

TLS provide sites of immune cell maturation and interactions linked to improved survival.^44^ Furthermore, BCR diversity and clonality affect tumour antigen-specific vs. broad antitumour responses, reflecting diverse functions.^45,49,55,56^

Enriched humoral signatures, particularly memory B cells, BCR abundance and diversity, and immunoglobulin gene rearrangement were associated with improved PFS and OS at baseline and on treatment in patients treated with ICIs.^71,73,77,80,81,83^ Heightened clonally expanded and memory B cells, at baseline and on treatment, are correlated with better ICI response.^52,53,70,71,73–78,82^ TLS-associated B cells interact with T cells, promoting effective antitumour immunity and ICI efficacy.^44,53,75,79,82^ However, two cases ascribe immunosuppressive roles to B cells in ICI response.^65,68^

Two studies reported contrasting findings of associations between B-cell decline on treatment and toxicity onset.^72,84^ One study found that on treatment B-cell decline was correlated with several irAEs,^72^ whereas another study reported no B-cell change in patients who developed an irAE of inflammatory arthritis,^84^ possibly reflecting distinct B-cell-driven mechanisms to this irAE. However, both studies reported proportional increases in specific B-cell subsets on treatment linked to the onset of irAEs.^72,84^ These may also reflect the impact of different ICI treatments, namely combination anti-PD-1/CTLA-4 vs. anti-PD-1 monotherapy.^72,84^

Four studies comparing different ICI regimens suggest that B-cell signatures may be more prognostically relevant in monotherapy vs. combination treatment,^72,79,81,84^ and could reflect more frequent irAEs in combination therapy. Future research should assess ICI-specific influences on B cells, and their value as predictive tools in ICI and relative to targeted therapies.

Th2-skewed immune response bias in the TME dominated by immunosuppressive cytokines (e.g. IL-10, VEGF and TGF-β) is consistent with class-switching of already primed B-cell infiltrates in favour of anti-inflammatory antibody isotypes such as IgG4.^88,89,99^ Class-switching to IgG4 and IgA, in patient circulation and tumours, is associated with less-favourable survival.^29,30,88–90^ A bias in favour of the less immunoactive IgG4 reflects an impaired, immunosuppressive humoral compartment,^88,89^ something reported in colorectal, oesophageal and other cancers.^28,100,101^

Correlations between autoantibodies against specific antigens (e.g. thyroid and collagen) and ICI-triggered irAE type (thyroid dysfunction, inflammatory arthritis) suggest direct associations between autoantibody induction and toxicity.^84,97^ Low baseline autoantibody levels and increased autoantibodies on treatment are associated with irAE onset, and high autoantibody levels at baseline are correlated with fewer irAEs, suggesting protective roles.^98^ Furthermore, several autoantibodies expressed in patients with active disease suggest a possible avenue to identify biomarkers of disease progression.^30^

Common limitations of the reviewed studies include small sample sizes, limited clinical and pathological information (particularly in public datasets) and technical challenges in *ex vivo* B-cell culture and stimulation, resulting in limited functional and mechanistic studies, critical for future research. Our study was restricted to English-language publications, based on practical and language translation limitations, and to ensure consistent interpretation of findings, which may have introduced bias but can be expanded in future work.

Overall, the increased frequency of memory B cells, particularly in tumours, is broadly associated with improved outcomes and better immunotherapy responses. Less-mature, alternatively activated and immunosuppressive circulating and tumour-resident B cells probably contribute to immune evasion. Class-switched, yet skewed, immunoglobulin expression favouring IgG4 and IgA is associated with worse outcomes. B cells produce tumour- and autoantigen-reactive antibodies, and an increase in autoantibodies in patients receiving ICI therapy is often associated with irAEs. Humoral immunity remains underexplored, where most studies assessing treatment responses and biomarker investigations are retrospective and observational. Larger prospective studies should fully evaluate antibodies as early detection biomarkers and the prognostic value of B cells to inform and improve immunotherapy responses.

## Supplementary Material

ljag074_Supplementary_Data

## Data Availability

All data are incorporated in the article and Supporting Information.
