# Supplementary material for: Phenotypic, functional, prognostic and predictive significance of B-cell and antibody responses in human melanoma: a scoping review
Source: Br J Dermatol. 2026 Feb 26;194(6):1046–63. doi: 10.1093/bjd/ljag074 (PMC13185749; doi:10.1093/bjd/ljag074)
Supplement: ljag074_Supplementary_Data [file ljag074_supplementary_data.docx]

Supplementary Table 1. Completed PRISMA-ScR (Preferred Reporting Items for Systematic reviews and Meta-Analyses extension for Scoping Reviews) Checklist

| **SECTION** | **ITEM** | **PRISMA-ScR CHECKLIST ITEM** | **REPORTED ON PAGE #** |
| --- | --- | --- | --- |
| **TITLE** | | | |
| Title | 1 | Identify the report as a scoping review. | 1 |
| **ABSTRACT** | | | |
| Structured summary | 2 | Provide a structured summary that includes (as applicable): background, objectives, eligibility criteria, sources of evidence, charting methods, results, and conclusions that relate to the review questions and objectives. | 4 |
| **INTRODUCTION** | | | |
| Rationale | 3 | Describe the rationale for the review in the context of what is already known. Explain why the review questions/objectives lend themselves to a scoping review approach. | 5 |
| Objectives | 4 | Provide an explicit statement of the questions and objectives being addressed with reference to their key elements (e.g., population or participants, concepts, and context) or other relevant key elements used to conceptualize the review questions and/or objectives. | 5 |
| **METHODS** | | | |
| Protocol and registration | 5 | Indicate whether a review protocol exists; state if and where it can be accessed (e.g., a Web address); and if available, provide registration information, including the registration number. | 4,6 |
| Eligibility criteria | 6 | Specify characteristics of the sources of evidence used as eligibility criteria (e.g., years considered, language, and publication status), and provide a rationale. | 5,6 |
| Information sources* | 7 | Describe all information sources in the search (e.g., databases with dates of coverage and contact with authors to identify additional sources), as well as the date the most recent search was executed. | 6 |
| Search | 8 | Present the full electronic search strategy for at least 1 database, including any limits used, such that it could be repeated. | 6, Supplementary Table 2, Figure 1 |
| Selection of sources of evidence† | 9 | State the process for selecting sources of evidence (i.e., screening and eligibility) included in the scoping review. | 6 |
| Data charting process‡ | 10 | Describe the methods of charting data from the included sources of evidence (e.g., calibrated forms or forms that have been tested by the team before their use, and whether data charting was done independently or in duplicate) and any processes for obtaining and confirming data from investigators. | 6 |
| Data items | 11 | List and define all variables for which data were sought and any assumptions and simplifications made. | 6 |
| Critical appraisal of individual sources of evidence§ | 12 | If done, provide a rationale for conducting a critical appraisal of included sources of evidence; describe the methods used and how this information was used in any data synthesis (if appropriate). | 6, Supplementary Table 4,5,6 and 7 |
| Synthesis of results | 13 | Describe the methods of handling and summarizing the data that were charted. | 5-7 |
| **RESULTS** | | | |
| Selection of sources of evidence | 14 | Give numbers of sources of evidence screened, assessed for eligibility, and included in the review, with reasons for exclusions at each stage, ideally using a flow diagram. | 5-7, Figure 1 |
| Characteristics of sources of evidence | 15 | For each source of evidence, present characteristics for which data were charted and provide the citations. | Supplementary Table 8 |
| Critical appraisal within sources of evidence | 16 | If done, present data on critical appraisal of included sources of evidence (see item 12). | Supplementary Tables 4,5,6 and 7 |
| Results of individual sources of evidence | 17 | For each included source of evidence, present the relevant data that were charted that relate to the review questions and objectives. | Supplementary Tables 8, 9, 10, 11 and 12 |
| Synthesis of results | 18 | Summarize and/or present the charting results as they relate to the review questions and objectives. | 7-12 |
| **DISCUSSION** | | | |
| Summary of evidence | 19 | Summarize the main results (including an overview of concepts, themes, and types of evidence available), link to the review questions and objectives, and consider the relevance to key groups. | 12-14 |
| Limitations | 20 | Discuss the limitations of the scoping review process. | 14 |
| Conclusions | 21 | Provide a general interpretation of the results with respect to the review questions and objectives, as well as potential implications and/or next steps. | 14 |
| **FUNDING** | | | |
| Funding | 22 | Describe sources of funding for the included sources of evidence, as well as sources of funding for the scoping review. Describe the role of the funders of the scoping review. | 2 |

Supplementary Table 2. Search Strategy followed in the search engines PubMed, Scopus and OVID Medline on 9^th^ October 2024

|  | Search terms |
| --- | --- |
| Restrictions | Humans  Full-text  English  Adult  From 2000-2024 |
| #1 | “Melanoma” [Title/Abstract] OR “Malignant melanoma” [Title/Abstract] OR Metastatic melanoma [Title/Abstract] OR “cutaneous melanoma” [Title/Abstract] |
| #2 | “B cell” [Title/Abstract] OR “B lymphocyte” [Title/Abstract] OR “B-cell” [Title/Abstract] OR “B-lymphocyte” [Title/Abstract] |
| #3 | #1 AND #2 |
| #4 | “B cell” [Title/Abstract] OR “melanoma” [Title/Abstract] OR “immunoglobulin” [Title/Abstract] OR “antibody” [Title/Abstract] OR “antibod” [Title/Abstract] OR “Ig” [Title/Abstract] OR “serum Ig” [Title/Abstract] OR “serum immunoglobulin” [Title/Abstract] OR “IgG” [Title/Abstract] OR “class switch” [Title/Abstract] OR “class-switch” [Title/Abstract] OR “immunoglobulin isotype” [Title/Abstract] OR “plasma cell” [Title/Abstract] OR “plasmablast” [Title/Abstract] OR “naïve B cell” [Title/Abstract] OR “regulatory B cell” [Title/Abstract] OR “regulatory B cell” [Title/Abstract] OR “double negative B cell” [Title/Abstract] OR “Breg” [Title/Abstract] OR “double-negative” [Title/Abstract] OR “atypical B cell” [Title/Abstract] OR “IgG” [Title/Abstract] OR “IgA” [Title/Abstract] OR “IgM” [Title/Abstract] OR “IgE” [Title/Abstract] OR “IgD” [Title/Abstract] OR “subclass” [Title/Abstract] OR “TLS” [Title/Abstract] OR “tertiary lymphoid structure” [Title/Abstract] OR “tumour-infiltrating B cell” [Title/Abstract] OR “TIL-B” [Title/Abstract] OR “melanoma immunotherapy” [Title/Abstract] OR “checkpoint inhibitor” [Title/Abstract] OR “immunotherapy” [Title/Abstract] OR “checkpoint blockade” [Title/Abstract] OR “anti-PD1” [Title/Abstract] OR “anti-CTLA4” [Title/Abstract] OR “treatment response” [Title/Abstract] |
| #5 | # 3AND #4 |

Supplementary Table 3. Search results produced from each search engine on the 9^th^ October 2024

| Search Engine | Results |
| --- | --- |
| PubMed | 827 |
| Scopus | 2759 |
| OVID Medline | 1081 |
| Total | **4667** |

Supplementary Table 4. JBI-approved checklists used to assess the quality of studies

| Type of Study | JBI-approved Checklist Item |
| --- | --- |
| Case control studies | Were the groups comparable other than the presence of disease in cases or the absence of disease in controls? |
|  | Were cases and controls matched appropriately? |
|  | Were the same criteria used for identification of cases and controls? |
|  | Was exposure measured in a standard, valid and reliable way? |
|  | Was exposure measured in the same way for cases and controls? |
|  | Were confounding factors identified? |
|  | Were strategies to deal with confounding factors stated? |
|  | Were outcomes assessed in a standard, valid and reliable way for cases and controls? |
|  | Was the exposure period of interest long enough to be meaningful? |
|  | Was appropriate statistical analysis used? |
| Cohort studies | Were the two groups similar and recruited from the same population? |
|  | Were the exposures measured similarly to assign people to both exposed and unexposed groups? |
|  | Was the exposure measured in a valid and reliable way? |
|  | Were confounding factors identified? |
|  | Were strategies to deal with confounding factors stated? |
|  | Were the groups/participants free of the outcome at the start of the study (or at the moment of exposure)? |
|  | Were the outcomes measured in a valid and reliable way? |
|  | Was the follow up time reported and sufficient to be long enough for outcomes to occur? |
|  | Was follow up complete, and if not, were the reasons to loss to follow up described and explored? |
|  | Were strategies to address incomplete follow up utilized? |
|  | Was appropriate statistical analysis used? |
| Cross sectional studies | Were the criteria for inclusion in the sample clearly defined? |
|  | Were the study subjects and the setting described in detail? |
|  | Was the exposure measured in a valid and reliable way? |
|  | Were objective, standard criteria used for measurement of the condition? |
|  | Were confounding factors identified? |
|  | Were strategies to deal with confounding factors stated? |
|  | Were the outcomes measured in a valid and reliable way? |
|  | Was appropriate statistical analysis used? |

Quality of studies was determined by the percentage of items that scored yes from the JBI checklists. Most studies were found to be of high quality (80-100%. green), followed by moderate quality (50-80%, orange). No studies were found to be low quality (<50%). For the quality assessment results, for each question studies may either be yes (Y), no (N), unclear (U) or not applicable (NA).

Supplementary Table 5. Quality assessment of case control studies using the Joanna Brigg’s Institute (JBI) checklist

| Case Control Studies | JBI-approved checklist item | | | | | | | | | | |
| --- | --- | --- | --- | --- | --- | --- | --- | --- | --- | --- | --- |
|  | **1** | **2** | **3** | **4** | **5** | **6** | **7** | **8** | **9** | **10** | **Score** |
| Andres 2005 | Y | U | Y | Y | Y | N | N | Y | Y | Y | **8** |
| Brase 2021 | Y | Y | Y | Y | Y | Y | Y | Y | Y | Y | **10** |
| Carpenter 2009 | Y | N | Y | Y | Y | N | N | Y | Y | Y | **7** |
| Crescioli 2023 | Y | N | Y | Y | Y | N | N | Y | Y | Y | **7** |
| De Jonge 2020 | Y | Y | Y | Y | Y | U | N | Y | Y | Y | **8** |
| Gadeyne 2021 | Y | Y | Y | Y | Y | Y | Y | Y | Y | Y | **10** |
| Harris 2022 | Y | Y | Y | Y | Y | Y | Y | Y | Y | Y | **10** |
| Karagiannis 2013 | Y | Y | Y | Y | Y | Y | N | Y | Y | Y | **9** |
| Karagiannis 2015 | Y | Y | Y | Y | Y | Y | Y | Y | Y | Y | **10** |
| Martinez-Escribano 2003 | Y | Y | Y | Y | Y | U | N | Y | Y | Y | **8** |
| Saul 2016 | Y | Y | Y | Y | Y | N | U | Y | Y | Y | **8** |
| Schina 2023 | Y | Y | Y | Y | Y | Y | N | Y | Y | Y | **9** |
| Valpione 2022 | Y | Y | Y | Y | Y | Y | U | Y | Y | Y | **9** |
| Versluis 2024 | Y | Y | Y | Y | Y | Y | Y | Y | Y | Y | **10** |
| Zhou 2024 | Y | Y | Y | Y | Y | Y | U | Y | Y | Y | **9** |

Supplementary Table 6. Quality assessment of cohort studies using the Joanna Brigg’s Institute (JBI) checklist

| Cohort Studies | JBI-approved checklist item | | | | | | | | | | | | |
| --- | --- | --- | --- | --- | --- | --- | --- | --- | --- | --- | --- | --- | --- |
|  | **1** | **2** | **3** | **4** | **5** | **6** | **7** | **8** | **9** | **10** | **11** | **Score** |  |
| Aklilu 2004 | Y | N | Y | N | N | Y | Y | Y | Y | N | Y | **7** |  |
| Das 2017 | Y | Y | Y | N | N | Y | Y | Y | N | N | Y | **7** |  |
| Kessler 2019 | Y | Y | Y | Y | Y | Y | Y | Y | Y | NA | Y | **10** |  |
| Mulder 2022 | Y | Y | Y | Y | N | Y | Y | Y | Y | N | Y | **9** |  |
| Ghosh 2022 | Y | Y | Y | Y | Y | Y | Y | Y | Y | Y | Y | **11** |  |
| Griss 2020 | Y | Y | Y | Y | Y | Y | Y | Y | Y | N | Y | **10** |  |

Supplementary Table 7. Quality assessment of cross-sectional studies using the Joanna Brigg’s Institute (JBI) checklist

| Cross Sectional Studies | JBI checklist item | | | | | | | | |
| --- | --- | --- | --- | --- | --- | --- | --- | --- | --- |
|  | **1** | **2** | **3** | **4** | **5** | **6** | **7** | **8** | **Score** |
| Anagnostou 2020 | Y | Y | Y | Y | Y | Y | Y | Y | **8** |
| Attrill 2022 | Y | Y | Y | Y | Y | N | Y | Y | **7** |
| Bosisio 2016 | Y | Y | Y | Y | Y | U | Y | Y | **7** |
| Cabrita 2020 | Y | Y | Y | Y | Y | U | Y | Y | **7** |
| Chen 2021 | Y | Y | Y | Y | Y | N | Y | Y | **7** |
| Cippioni 2012 | Y | Y | Y | Y | N | N | Y | N | **5** |
| Damsky 2019 | Y | Y | Y | Y | Y | N | Y | Y | **7** |
| De Moel 2019 | Y | Y | Y | Y | N | N | Y | Y | **6** |
| Diem 2019 | Y | Y | Y | Y | Y | U | Y | Y | **7** |
| Ding 2023 | Y | Y | Y | Y | Y | U | Y | Y | **7** |
| Dollinger 2020 | Y | Y | Y | Y | N | N | Y | Y | **6** |
| Edmonds 2022 | Y | Y | Y | Y | Y | U | Y | Y | **6** |
| Egan 2023 | Y | Y | Y | Y | U | N | Y | Y | **6** |
| Erdag 2012 | Y | Y | Y | Y | Y | Y | Y | Y | **8** |
| Freeman 2022 | Y | Y | Y | Y | Y | Y | Y | Y | **8** |
| García-Mulero 2021 | Y | Y | Y | Y | Y | Y | Y | Y | **8** |
| Garg 2016 | Y | Y | Y | Y | Y | Y | Y | Y | **8** |
| Gatto 2023 | Y | Y | Y | Y | Y | Y | Y | Y | **8** |
| Gilbert 2011 | Y | Y | Y | Y | Y | Y | Y | Y | **8** |
| Gorris 2022 | Y | Y | Y | Y | Y | Y | Y | Y | **8** |
| Griss 2020 | Y | N | Y | Y | Y | Y | Y | Y | **7** |
| Helmink 2020 | Y | Y | Y | Y | Y | Y | Y | Y | **8** |
| Hillen 2007 | Y | Y | Y | Y | Y | Y | Y | Y | **8** |
| Hoch 2022 | Y | Y | Y | Y | Y | U | Y | Y | **7** |
| Huang 2023 | Y | Y | Y | Y | N | N | Y | Y | **6** |
| Iglesia 2016 | Y | N | Y | Y | Y | Y | Y | Y | **7** |
| Imahashi 2022 | Y | Y | Y | Y | Y | Y | Y | Y | **8** |
| Kang 2020 | Y | Y | Y | Y | Y | Y | Y | Y | **8** |
| Karagiannis 2022 | Y | N | Y | Y | Y | Y | Y | Y | **7** |
| Ladanyi 2011 | Y | Y | Y | Y | Y | Y | Y | Y | **8** |
| Ladanyi 2014 | Y | Y | Y | Y | Y | N | Y | Y | **7** |
| Lardone 2016 | Y | Y | Y | Y | Y | Y | Y | Y | **8** |
| Li 2023 | Y | Y | Y | Y | Y | U | Y | Y | **7** |
| Liu 2021 | Y | Y | Y | Y | Y | U | Y | Y | **7** |
| Lundberg 2021 | Y | Y | Y | Y | U | U | Y | Y | **6** |
| Lynch 2021 | Y | Y | Y | Y | Y | Y | Y | Y | **8** |
| Martinez-Rodriquez 2014 | Y | Y | Y | U | U | Y | Y | Y | **6** |
| Mastracci 2020 | Y | Y | Y | Y | Y | Y | Y | Y | **8** |
| Oneiva 2022 | Y | Y | Y | Y | Y | N | Y | Y | **7** |
| Pourmaleki 2022 | Y | Y | Y | Y | Y | U | Y | Y | **7** |
| Quek 2024 | Y | Y | Y | Y | Y | N | Y | Y | **7** |
| Selitsky 2019 | Y | Y | Y | Y | Y | Y | Y | Y | **8** |
| Somasundaram 2017 | Y | Y | Y | Y | U | N | Y | Y | **6** |
| Song 2023 | Y | Y | Y | Y | Y | Y | Y | Y | **8** |
| Stowman 2018 | Y | Y | Y | Y | U | N | Y | Y | **6** |
| Therien 2022 | Y | Y | Y | Y | Y | N | Y | Y | **7** |
| Van de veen 2020 | Y | Y | Y | Y | Y | U | Y | Y | **7** |
| Varn 2018 | Y | Y | Y | Y | Y | N | Y | Y | **7** |
| Wang 2022 | Y | Y | Y | Y | Y | U | Y | Y | **7** |
| Werner 2021 | Y | Y | Y | Y | U | N | Y | Y | **6** |
| Werner 2021 | Y | Y | Y | Y | U | U | Y | Y | **6** |
| Wu 2020 | Y | Y | Y | Y | Y | U | Y | Y | **7** |
| Wu 2022 | Y | Y | Y | Y | Y | Y | Y | Y | **8** |
| Xiong 2019 | Y | Y | Y | Y | Y | U | Y | Y | **7** |
| Yan 2019 | Y | Y | Y | Y | U | U | Y | Y | **6** |
| Yan 2020 | Y | Y | Y | Y | Y | Y | Y | Y | **8** |
| Yu 2022 | Y | Y | Y | Y | Y | U | Y | Y | **7** |
| Zhang 2024 | Y | Y | Y | Y | Y | Y | Y | Y | **8** |
| Zhao 2022 | Y | Y | Y | Y | Y | U | Y | Y | **7** |

Supplementary Table 8. Summary Data of all included studies investigating B cell-related or antibody-related responses in patients with melanoma.

| Author | Title | DOI | Year of publication | Study Focus | Study Design | Techniques Used | Type of Melanoma |
| --- | --- | --- | --- | --- | --- | --- | --- |
| Aklilu | Depletion of normal B cells with rituximab as an adjunct to IL-2 therapy for renal cell carcinoma and melanoma | 10.1093/annonc/mdh280 | 2004 | Response to treatment | Cohort study | Flow cytometry | Metastatic cutaneous melanoma |
| Anagnostou | Integrative Tumour and Immune Cell Multi-omic Analyses Predict Response to Immune Checkpoint Blockade in Melanoma. | 10.1016/j.xcrm.2020.100139 | 2020 | Response to treatment | Cross sectional | WES, RNA-Seq, TCR-Seq | Metastatic cutaneous melanoma |
| Andrés | Prognostic value of quantitative immune alterations in melanoma patients | 10.4321/S0378-48352006000100004 | 2005 | Prognosis and survival | Case control | Flow cytometry | Cutaneous melanoma |
| Attrill | Detailed spatial immunophenotyping of primary melanomas reveals immune cell subpopulations associated with patient outcome. | 10.3389/fimmu.2022.979993 | 2022 | Prognosis and survival | Cross sectional | Flow cytometry, multiplex immunohistochemistry, multispectral imaging | Primary cutaneous melanoma |
| Bosisio | Plasma cells in primary melanoma. Prognostic significance and possible role of IgA | 10.1038/modpathol.2016.28 | 2016 | Prognosis and survival | Cross sectional | Immunohistochemistry, gene scan analysis | Invasive primary cutaneous melanoma |
| Brase | Role of Tumour-Infiltrating B Cells in Clinical Outcome of Patients with Melanoma Treated with Dabrafenib Plus Trametinib. | 10.1158/1078-0432.CCR-20-3586 | 2021 | Prognosis and survival | Case control | Gene expression profiling (GEP) using NanoString technology, Immunohistochemistry | BRAF V600-mutant metastatic cutaneous melanoma (Stage IIIC/IV). |
| Cabrita | Tertiary lymphoid structures improve immunotherapy and survival in melanoma. | 10.1038/s41586-019-1914-8 | 2020 | Prognosis and survival | Cross sectional | Immunohistochemistry, RNA sequencing and microarray, High-plex proteomic analysis | Primary and metastatic cutaneous melanoma |
| Carpenter | Collapse of the CD27+ B-cell compartment associated with systemic plasmacytosis in patients with advanced melanoma and other cancers. | 10.1158/1078-0432.CCR-09-0537 | 2009 | Phenotype and function | Case control | Flow cytometry, stimulation assays | Cutaneous melanoma |
| Chen | Spatiotemporal Analysis of B Cell- and Antibody Secreting Cell-Subsets in Human Melanoma Reveals Metastasis-, Tumour Stage-, and Age-Associated Dynamics. | 10.3389/fcell.2021.677944 | 2021 | Phenotype and function | Cross sectional | Seven-Colour Multiplex Immunohistochemistry | Cutaneous melanoma (primary tumours and metastatic samples) |
| Cipponi | Neogenesis of lymphoid structures and antibody responses occur in human melanoma metastases. | 10.1158/0008-5472.CAN-12-1377 | 2012 | Antibody | Cross sectional | Immunohistochemistry, qRT-PCR | Metastatic cutaneous melanoma |
| Crescioli | B cell profiles, antibody repertoire and reactivity reveal dysregulated responses with autoimmune features in melanoma. | 10.1038/s41467-023-39042-y | 2023 | Phenotype and function | Case control | Mass cytometry, multi-colour flow cytometry and cell sorting, immunofluorescence, single cell RNAseq, visium spatial transcriptomics, antibody cloning/production and immuno mass spectrometry. | Cutaneous melanoma |
| Damsky | B cell depletion or absence does not impede anti-tumour activity of PD-1 inhibitors. | 10.1186/s40425-019-0613-1 | 2019 | Prognosis and survival | Cross sectional | Immunofluorescence | Cutaneous and mucosal melanoma |
| Das | Early B cell changes predict autoimmunity following combination immune checkpoint blockade | 10.1172/JCI96798 | 2017 | Response to treatment | Cohort study | Flow cytometry | Metastatic cutaneous melanoma |
| de Jonge | Inflammatory B cells correlate with failure to checkpoint blockade in melanoma patients | 10.1080/2162402X.2021.1873585 | 2020 | Phenotype and function | Case control | Flow cytometry, RNA seq | Cutaneous melanoma |
| De Moel | Autoantibody development under treatment with immune- checkpoint inhibitors | 10.1158/ 2326-6066.CIR-18-0245 | 2019 | Antibody | Cross sectional | Immunofluorescence assays, fluorescence enzyme immunoassays, chemiluminescent immunoassays | Metastatic cutaneous melanoma |
| Diem | Immunoglobulin G and Subclasses as Potential Biomarkers in Metastatic Melanoma Patients Starting Checkpoint Inhibitor Treatment. | 10.1097/CJI.0000000000000255 | 2019 | Antibody | Cross sectional | Immunoturbidimetric methods | Metastatic cutaneous melanoma |
| Ding | Antigen presentation by clonally diverse CXCR5+ B cells to CD4 and CD8 T cells is associated with durable response to immune checkpoint inhibitors. | 10.1136/jitc-2022-005644 | 2023 | Response to treatment | Cross sectional | RNAseq | Cutaneous melanoma |
| Dollinger | Divergent resistance mechanisms to immunotherapy explain responses in different skin cancers | 10.3390/cancers12102946 | 2020 | Response to treatment | Cross sectional | Single cell RNAseq | Metastatic cutaneous melanoma |
| Edmonds | Tertiary lymphoid structures in desmoplastic melanoma have increased lymphocyte density, lymphocyte proliferation, and immune cross talk with tumour when compared to non-desmoplastic melanomas | 10.1080/2162402X.2022.2164476 | 2022 | Phenotype and function | Cross sectional | Multiplex Immunofluorescence Histology | Primary Desmoplastic Melanoma (PDM) Non-Desmoplastic Metastatic Melanoma (NDMM) |
| Egan | Small Gene Networks Delineate Immune Cell States and Characterize Immunotherapy Response in Melanoma | 10.1158/2326-6066.CIR-22-0563 | 2023 | Response to treatment | Cross sectional | Bulk and single cell RNAseq | Metastatic cutaneous melanoma |
| Erdag | Immunotype and immunohistologic characteristics of tumour-infiltrating immune cells are associated with clinical outcome in metastatic melanoma. | 10.1158/0008-5472.CAN-11-3218 | 2012 | Phenotype and function | Cross sectional | Immunohistochemistry | Metastatic cutaneous melanoma |
| Freeman | Combined tumour and immune signals from genomes or transcriptomes predict outcomes of checkpoint inhibition in melanoma. | 10.1016/j.xcrm.2021.100500 | 2022 | Prognosis and survival | Cross sectional | RNAseq | Cutaneous, uveal and vulval melanoma |
| Gadeyne | A Multi-Omics Analysis of Metastatic Melanoma Identifies a Germinal Centre-Like Tumour Microenvironment in HLA-DR-Positive Tumour Areas | 10.1126/sciadv.aaz3559 | 2021 | Phenotype and function | Case control | Multiplex immunohistochemistry, RNAseq, Luminex. | Metastatic cutaneous melanoma |
| García-Mulero | Additive role of immune system infiltration and angiogenesis in uveal melanoma progression | 10.3389/fonc.2021.636057 | 2021 | Prognosis and survival | Cross sectional | RNAseq | Uveal melanoma |
| Garg | Tumour-associated B cells in cutaneous primary melanoma and improved clinical outcome | 10.1016/j.humpath.2016.03.022 | 2016 | Prognosis and survival | Cross sectional | Immunohistochemistry Quantitative Digital Imaging RNAseq | Cutaneous primary melanoma, excluding desmoplastic melanomas |
| Gatto | Early Increase of Circulating Transitional B Cells and Autoantibodies to Joint-Related Proteins in Patients with Metastatic Melanoma Developing Checkpoint Inhibitor-Induced Inflammatory Arthritis | 10.1002/art.42406 | 2023 | Response to treatment | Cross sectional | Flow cytometry, autoantibody detection | Metastatic cutaneous melanoma |
| Ghosh | Lower baseline autoantibody levels are associated with immune-related adverse events from immune checkpoint inhibition | 10.1136/jitc-2021-004008 | 2022 | Antibody | Cohort study | Autoantigen array, immunofluorescence, ELISA | Metastatic cutaneous melanoma |
| Gilbert | Monitoring the systemic human memory B cell compartment of melanoma patients for anti-tumour IgG antibodies. | 10.1371/journal.pone.0019330 | 2011 | Antibody | Cross sectional | ELISA on peripheral blood | Cutaneous melanoma (stages I - IV) |
| Gorris | Paired primary and metastatic lesions of patients with ipilimumab-treated melanoma: high variation in lymphocyte infiltration and HLA-ABC expression whereas tumor mutational load is similar and correlates with clinical outcome | 10.1136/jitc-2021-004329 | 2022 | Phenotype and function | Cross sectional | Bulk RNAseq, mIHC | Primary and metastatic cutaneous melanoma |
| Griss | ReactomeGSA - Efficient Multi-Omics Comparative Pathway Analysis. | 10.1074/mcp.TIR120.002155 | 2020 | Phenotype and function | Cross sectional | Multi-omics datasets: scRNA-seq, bulk RNA-seq, microarray, proteomics | Cutaneous melanoma |
| Griss | B cells sustain inflammation and predict response to immune checkpoint blockade in human melanoma | 10.1038/s41467-019-12160-2 | 2019 | Phenotype and function | Cohort study | Induction experiments, flow cytometry, cell viability assays, proteomic analysis and transcriptomic analysis, IHC. | Metastatic cutaneous melanoma |
| Harris | Enriched circulating and tumor-resident TGF-β(+) regulatory B cells in patients with melanoma promote FOXP3(+) Tregs | 10.1080/2162402X.2022.2104426 | 2022 | Phenotype and function | Case control | Flow cytometry, CyTOF, immunofluorescence, single cell RNAseq, and B:T cell co-culture | Metastatic cutaneous melanoma |
| Helmink | B cells and tertiary lymphoid structures promote immunotherapy response | 10.1038/s41586-019-1922-8 | 2020 | Prognosis and survival | Cross sectional | RNAseq | Cutaneous melanoma |
| Hillen | Leukocyte infiltration and tumor cell plasticity are parameters of aggressiveness in primary cutaneous melanoma | 10.1007/s00262-007-0353-9 | 2007 | Prognosis and survival | Cross sectional | Immunohistochemistry | Primary cutaneous melanoma |
| Hoch | Multiplexed imaging mass cytometry of the chemokine milieus in melanoma characterizes features of the response to immunotherapy | 10.1126/sciimmunol.abk1692 | 2022 | Phenotype and function | Cross sectional | Imaging mass cytometry, single cell RNAseq | Stage III and IV metastatic cutaneous melanoma |
| Huang | A BRAF mutation-associated gene risk model for predicting the prognosis of melanoma | 10.1016/j.heliyon.2023.e15939 | 2023 | Prognosis and survival | Cross Sectional | RNAseq | Cutaneous melanoma |
| Iglesia | Genomic Analysis of Immune Cell Infiltrates Across 11 Tumor Types. | 10.1093/jnci/djw144 | 2016 | Prognosis and survival | Cross sectional | Single cell RNAseq | Cutaneous melanoma |
| Imahashi | Activated B cells suppress T-cell function through metabolic competition. | 10.1007/s00262-020-02686-6 | 2022 | Phenotype and function | Cross Sectional | Cell culture, flow cytometry, mass cytometry, bulk and single cell RNAseq. | Cutaneous melanoma |
| Kang | Significance of Tumor Mutation Burden in Immune Infiltration and Prognosis in Cutaneous Melanoma | 10.3389/fonc.2020.573141 | 2020 | Prognosis and survival | Cross sectional | RNAseq, somatic mutation data analysis | Cutaneous melanoma |
| Karagiannis | Elevated IgG4 in patient circulation is associated with the risk of disease progression in melanoma | 10.1080/2162402X.2015.1032492 | 2015 | Antibody | Case control | Luminex, immunohistochemistry, flow cytometry | Metastatic cutaneous melanoma |
| Karagiannis | Innate stimulation of B cells ex vivo enhances antibody secretion and identifies tumour-reactive antibodies from cancer patients. | 10.1093/cei/uxab005 | 2022 | Phenotype and function | Cross sectional | Proliferation assay, Flow cytometry, ELISA | Cutaneous melanoma |
| Karagiannis | IgG4 subclass antibodies impair antitumor immunity in melanoma | 10.1172/JCI65579 | 2013 | Antibody | Case control | Ex vivo stimulation assays, RNA and protein extraction, western blots, ELISA, qtPCR, immunohistochemistry, Luminex, flow cytometry, image stream, ADCC/ADCP, antibody engineering | Cutaneous melanoma |
| Kessler | Serum IgG is associated with risk of melanoma in the Swedish Amoris study | 10.3389/fonc.2019.01095 | 2019 | Antibody | Cohort | Turbidimetric assay to measure serum Ig | Cutaneous melanoma |
| Ladanyi | Prognostic impact of B-cell density in cutaneous melanoma. | 10.1007/s00262-011-1071-x | 2011 | Phenotype and function | Cross sectional | Immunohistochemistry | Primary cutaneous melanoma |
| Ladanyi | Ectopic lymphoid structures in primary cutaneous melanoma. | 10.1007/s12253-014-9784-8 | 2014 | Phenotype and function | Cross sectional | Immunohistochemistry | Primary cutaneous melanoma |
| Lardone | Cross-platform comparison of independent datasets identifies an immune signature associated with improved survival in metastatic melanoma. | 10.18632/oncotarget.7361 | 2016 | Prognosis and survival | Cross sectional | RNAseq | Metastatic cutaneous melanoma |
| Li | The evaluation of tumor microenvironment infiltration and the identification of angiogenesis-related subgroups in skin cutaneous melanoma | 10.1007/s00432-023-04680-8 | 2023 | Phenotype and function | Cross sectional | RNAseq, microarray analysis, qtPCR | Cutaneous Melanoma |
| Liu | Identification of subtypes correlated with tumor immunity and immunotherapy in cutaneous melanoma | 10.1016/j.csbj.2021.08.005 | 2021 | Response to treatment | Cross sectional | Single cell RNAseq | Cutaneous melanoma |
| Lundberg | B cell-related gene signature and cancer immunotherapy response. | 10.1038/s41416-021-01674-6 | 2021 | Prognosis and survival | Cross sectional | RNAseq | Cutaneous melanoma |
| Lynch | Heterogeneity in tertiary lymphoid structure B-cells correlates with patient survival in metastatic melanoma | 10.1136/jitc-2020-002273 | 2021 | Prognosis and survival | Cross sectional | Multiplex immunohistochemistry and image analysis | Metastatic cutaneous melanoma |
| Martinez-Escribano | Changes in the number of CD80+, CD86+, and CD28+ peripheral blood lymphocytes have prognostic value in melanoma patients | 10.1016/S0198-8859(03)00122-8 | 2003 | Prognosis and survival | Case control | Flow cytometry | Primary cutaneous melanoma |
| Martinez-Rodriguez | A significant percentage of CD20-positive TILs correlates with poor prognosis in patients with primary cutaneous malignant melanoma. | 10.1111/his.12437 | 2014 | Prognosis and survival | Cross sectional | Immunohistochemistry | Primary cutaneous melanoma |
| Mastracci | Response to ipilimumab therapy in metastatic melanoma patients: potential relevance of CTLA-4+ tumor infiltrating lymphocytes and their in-situ localization | 10.1007/s00262-020-02494-y | 2020 | Response to treatment | Cross sectional | H&E staining, Immunohistochemistry (IHC), | Stage IV metastatic cutaneous melanoma, and mucosal melanoma |
| Mulder | Histopathological and immunological spectrum in response evaluation of talimogene laherparepvec treatment and correlation with durable response in patients with cutaneous melanoma | 10.1097/CMR.0000000000000824 | 2022 | Antibody | Cohort study | Immunohistochemistry, PCR | Cutaneous melanoma |
| Onieva | High IGKC-Expressing Intratumoral Plasma Cells Predict Response to Immune Checkpoint Blockade | 10.3390/ijms23169124 | 2022 | Response to treatment | Cross sectional | Single cell RNA seq, multispectral immunofluorescence and machine learning | Metastatic melanoma, including cutaneous, uveal and mucosal |
| Pourmaleki | Tumor MHC Class I Expression Associates with Intralesional IL2 Response in Melanoma | 10.1158/2326-6066.CIR-21-1083 | 2022 | Response to treatment | Cross sectional | Multiplexed immunofluorescence (IF)  Bulk and single cell RNA proteomics and transcriptomics Bulk DNA seq Immunohistochemistry (IHC) | In-transit cutaneous melanoma metastases |
| Quek | Single-cell spatial multiomics reveals tumor microenvironment vulnerabilities in cancer resistance to immunotherapy | 10.1016/j.celrep.2024.114392 | 2024 | Prognosis and survival | Cross sectional | scRNA-seq, CITE-seq: To measure both RNA and protein expression at the single-cell level. PhenoCycler (CODEX): High-plex tissue imaging to spatially resolve cell types and their interactions in the TME. | Metastatic cutaneous melanoma |
| Saul | IgG subclass switching and clonal expansion in cutaneous melanoma and normal skin | 10.1038/srep29736 | 2016 | Antibody | Case control | IHC, RNA extraction, qt-PCR, IgG sequence analysis, RNA seq | Metastatic cutaneous melanoma |
| Schina | Intratumoral T-cell and B-cell receptor architecture associates with distinct immune tumor microenvironment features and clinical outcomes of anti-PD-1/L1 immunotherapy | 10.1136/jitc-2023-006941 | 2023 | Prognosis and survival | Case control | Bioinformatics and machine learning analyses to large transcriptomic datasets of tumor biopsies - TCR and BCR, bulk-RNA-Seq | Metastatic cutaneous melanoma |
| Selitsky | Prognostic value of B cells in cutaneous melanoma. | 10.1186/s13073-019-0647-5 | 2019 | Prognosis and survival | Cross sectional | RNA seq and BCR repertoire analysis | Cutaneous melanoma |
| Somasundaram | Tumor-associated B-cells induce tumor heterogeneity and therapy resistance | 10.1038/s41467-017-00452-4 | 2017 | Response to treatment | Cross sectional | Co-culture, RNAseq Immunohistochemistry Flow Cytometry | Metastatic cutaneous melanoma |
| Song | Role of single cell ferroptosis regulation in intercellular communication and skin cutaneous melanoma progression and immunotherapy | 10.1007/s00262-023-03504-5 | 2023 | Phenotype and function | Cross sectional | scRNA-seq, Bulk RNA-seq | Cutaneous melanoma |
| Stowman | Lymphoid aggregates in desmoplastic melanoma have features of tertiary lymphoid structures | 10.1097/CMR.0000000000000439 | 2018 | Phenotype and function | Cross sectional | Immunohistochemistry | Desmoplastic melanoma (DM) |
| Therien | Spatial biology analysis reveals B cell follicles in secondary lymphoid structures may regulate anti-tumor responses at initial melanoma diagnosis. | 10.3389/fimmu.2022.952220 | 2022 | Prognosis and survival | Cross sectional | Flow Cytometry, NanoString Digital Spatial Profiling (DSP): | Metastatic cutaneous melanoma |
| Valpione | Tumour infiltrating B cells discriminate checkpoint blockade-induced responses. | 10.1016/j.ejca.2022.09.022 | 2022 | Response to treatment | Case control | Single-cell RNA-seq Surface protein expression (REAP-Seq) Bulk RNA sequencing B cell receptor (BCR) and T cell receptor (TCR) clonotype reconstruction | Metastatic cutaneous melanoma |
| van de Veen | A novel proangiogenic B cell subset is increased in cancer and chronic inflammation. | 10.3389/fimmu.2021.675146 | 2020 | Phenotype and function | Cross sectional | RNAseq, flow cytometry, bead suspension arrays, qPCR and confocal microscopy | Cutaneous melanoma |
| Varn | A B cell-derived gene expression signature associates with an immunologically active tumor microenvironment and response to immune checkpoint blockade therapy. | 10.1080/2162402X.2018.1513440 | 2018 | Response to treatment | Cross sectional | RNAseq | Cutaneous melanoma |
| Versluis | Interferon-gamma signature as prognostic and predictive marker in macroscopic stage III melanoma. | 10.1136/jitc-2023-008125 | 2024 | Prognosis and survival | Case control | Immunohistochemistry, RNA-Seq | Stage III metastatic cutaneous melanoma |
| Wang | Characterization of the treatment-naive immune microenvironment in melanoma with BRAF mutation | 10.1136/jitc-2021-004095 | 2022 | Prognosis and survival | Cross sectional | Single-cell RNA seq, bulk RNA seq, flow cytometry and immunohistochemistry (IHC). | BRAF-mutant and wild type metastatic melanoma |
| Werner | A Standardized Analysis of Tertiary Lymphoid Structures in Human Melanoma: Disease Progression- and Tumor Site-Associated Changes with Germinal Centre Alteration. | 10.3390/diagnostics11071238 | 2021 | Phenotype and function | Cross sectional (some patients longitudinal Cohort Study) | Multiplex immunohistochemistry | Primary and metastatic cutaneous melanoma |
| Werner | Loss of Lymphotoxin Alpha-Expressing Memory B Cells Correlates with Metastasis of Human Primary Melanoma. | 10.3389/fimmu.2023.1176994 | 2021 | Phenotype and function | Cross sectional | Immunohistochemistry | Primary cutaneous melanoma |
| Wu | CD20(+)CD22(+)ADAM28(+) B Cells in Tertiary Lymphoid Structures Promote Immunotherapy Response | 10.3389/fimmu.2022.865596 | 2022 | Prognosis and survival | Cross sectional | Single cell RNA seq, Bulk RNA seq, immunofluorescence, in vivo experiments and qRT-PCR | Cutaneous melanoma |
| Wu | PD-L1(+) regulatory B cells act as a T cell suppressor in a PD-L1-dependent manner in melanoma patients with bone metastasis. | 10.1016/j.molimm.2020.01.008 | 2020 | Phenotype and function | Cross sectional | Peripheral blood FACS, IFN-γ suppression assay | Cutaneous melanoma |
| Xiong | Prognostic value of the expression of chemokines and their receptors in regional lymph nodes of melanoma patients | 10.1111/jcmm.15015 | 2019 | Prognosis and survival | Cross sectional | RNAseq | Cutaneous melanoma, LN and metastases |
| Yan | Prognostic Role of Tumor Mutation Burden Combined with Immune Infiltrates in Skin Cutaneous Melanoma Based on Multi-Omics Analysis | 10.3389/fonc.2020.570654 | 2020 | Prognosis and survival | Cross sectional | Somatic mutation analysis | Cutaneous melanoma |
| Yan | Understanding heterogeneous tumor microenvironment in metastatic melanoma | 10.1371/journal.pone.0216485 | 2019 | Phenotype and function | Cross sectional | Multiplexed immunofluorescence imaging | Metastatic cutaneous melanoma |
| Yu | A Pan-Cancer Analysis of Tumor-Infiltrating B Cell Repertoires | 10.3390/ijms22052669 | 2022 | Phenotype and function | Cross sectional | Bulk RNA seq | Primary and metastatic cutaneous melanoma |
| Zhang | Comprehensive analysis of single cell and bulk RNA sequencing reveals the heterogeneity of melanoma tumor microenvironment and predicts the response of immunotherapy | 10.1007/s00011-024-01905-5 | 2024 | Response to treatment | Cross sectional | Single cell RNAseq | Cutaneous melanoma |
| Zhao | A multi-omics deep learning model for hypoxia phenotype to predict tumor aggressiveness and prognosis in uveal melanoma for rationalized hypoxia-targeted therapy | 10.3389/fimmu.2021.790119 | 2022 | Prognosis and survival | Cross sectional | RNAseq | Uveal melanoma |
| Zhou | The prognostic implications of cuproptosis-related gene signature and the potential of PPIC as a promising biomarker in cutaneous melanoma | 10.1111/pcmr.13185 | 2024 | Prognosis and survival | Case control | scRNAseq, Machine learning, In vitro functional assays: transwell migration, and invasion assays | Cutaneous melanoma |

Supplementary Table 9. Studies investigating Phenotype and Function of B cells in melanoma

| Author | Title | Year of publication | Aims of Study | Techniques Used | Patient Demographics | Key Findings |
| --- | --- | --- | --- | --- | --- | --- |
| Carpenter | Collapse of the CD27+ B-cell compartment associated with systemic plasmacytosis in patients with advanced melanoma and other cancers. | 2009 | Study whether B cell physiology is altered in the presence of melanoma and other cancers. | Flow cytometry | Patients with advanced melanoma (N=26), (N=14 with resected disease, N=12 with active disease), N=20 healthy volunteers | - Alterations in the CD27+ B cell compartment in patient blood compared to healthy volunteers, both resected and active patients had significantly lower percentages of CD27+ B cells among total CD19+ cells in peripheral blood. - The percentage of CD19+ cells expressing CD25 was significantly lower in AD patients compared to normal donors, and there was a significantly higher expression of CD54 for AD patients compared to NED patients or normal donors. - All other variables studied – including CD40, which critically regulates memory B cell formation – were not significantly different among CD19+ cells from donors in the three groups. |
| Chen | Spatiotemporal Analysis of B Cell- and Antibody Secreting Cell-Subsets in Human Melanoma Reveals Metastasis-, Tumor Stage-, and Age-Associated Dynamics. | 2021 | Study the role of tumor-associated B cells (TAB) and antibody-secreting cells (ASC) in human melanoma, examining their distribution, dynamics, and association with tumor progression, metastasis, and patient age. | Immunohistochemistry | Primary melanomas from patients (N=97) (N=53 without metastasis, N=44 with metastasis). Metastatic melanoma samples from patients (N=57) (N=16 locoregional, N=41 distant metastases). Age range from 19 to 93 years; mean age of 64. | - The study found that B cell subpopulations, particularly memory-like B cells and plasma cell-like ASC, exhibit dynamic changes during melanoma progression. - Enrichment of memory-like B cells at primary tumour sites and plasma cell-like antibody secreting cells in distant metastasis compared to primary tumours. - Memory-like B cells are associated with better outcomes, and were proportionally increased in primary tumours, while plasma cell-like ASC are enriched in distant metastases. - Patients above the median age of 68 years had significantly higher plasmablast-like ASC frequencies compared to younger patients. |
| Crescioli | B cell profiles, antibody repertoire and reactivity reveal dysregulated responses with autoimmune features in melanoma. | 2023 | Deep phenotyping of circulating and tumour-resident B cells and serum autoantibodies in melanoma patients. | Mass cytometry, multi-colour flow cytometry and cell sorting, immunofluorescence of tissue, transcriptomic analysis (single cell RNAseq, visium spatial, antibody repertoire analysis), antibody cloning/production and immune-mass spectrometry. | Blood collected from patients with cutaneous melanoma (N=35) and healthy volunteers (N=13) for CyTOF analysis and from patients for flow analysis (N=29). Blood for serum autoantibody analysis was collected from N=31 patients. All patients were immunotherapy naïve. | - CyTOF analysis compared B cell phenotypes of 35 patient’s vs 13 healthy volunteers. In melanoma patients, there were significantly greater plasmablasts, and decreased DN1 cells, non-switched memory and resting class switched memory. - IF staining of melanoma metastases showed that B cells were mainly localized at the tumour edge but also formed small clusters inside tumours and in peritumoral areas. - scRNAseq of matched blood and tumours showed a decrease in naive clusters and increase in memory in tumour compared to blood. Extracted variable and constant region genes from memory B cells from blood and tumours revealed a significant difference between both V, D, J gene combinations and usage between blood and tumour, suggesting distinct antibody responses. - Sequences from tumour-associated memory B cells also showed evidence of SHM, clonal expansion and CSR from IgG1 to IgG2. - There was also a significant increase of the expression of RAG1 and RAG2, genes known to be involved in B cell and T cell receptor rearrangements, in melanoma compared to normal skin. |
| de Jonge | Inflammatory B cells correlate with failure to checkpoint blockade in melanoma patients | 2020 | Study the phenotype and functionality of B cells from melanoma patients in response to different therapies. | Flow cytometry and RNA seq. | Cohort 1 melanoma patients (N=58), Cohort 2 melanoma patients treated with ipilimumab (N=22), Cohort 3 melanoma patients (N=5), Cohort 4 melanoma patients (N=10) | - Studied B cells in the blood at baseline. B cells were identified as CD19+ and their subsets like naïve, unswitched memory and switched memory B cells as IgD+CD27−, IgD+CD27+ and IgD−CD27+, respectively. A decrease in B cells was found in stage IV patients compared to HV. - A difference in unswitched memory B cells was observed, with higher frequencies in stage I/II compared to stage III and IV patients. - When comparing B cells from patients responding positively to Ipilimumab with the ones showing no benefit, patients not responding to treatment exhibited a higher expression of IL-6R and had a higher frequency of B cells producing TNFα and IL-6, but no significant differences between responders and non-responders when looking at frequencies of B cells subsets and expression of CXCR3, PD-1, Fas receptor, IL-10 and GMCSF. |
| Edmonds | Tertiary lymphoid structures in desmoplastic melanoma have increased lymphocyte density, lymphocyte proliferation, and immune cross talk with tumor when compared to non-desmoplastic melanomas | 2022 | Compares the presence, structure, and immune activity of TLSs in primary desmoplastic melanoma (PDM) and non-desmoplastic metastatic melanoma (NDMM). Evaluates the role of tumour-infiltrating immune cells to understand potential mechanisms of ICI therapy response. | Multiplex Immunofluorescence histology | PDM Cohort (N =11) with a median age of 67 years (88% Stage II, 12% Stage I) NDMM Cohort (N = 30, TLS-positive) with a median age of 56 years (57% Stage IIIB, 43% Stage IV) | - PDM tumours contain more immunologically active TLSs than NDMM (73% compared to 47%), with higher T and B cell densities and proliferation rates. - The intratumoral location of PDM TLSs may enhance immune crosstalk between TLS and tumour. These features may contribute to the high response rates of desmoplastic melanoma to immune checkpoint blockade (ICB). |
| Erdag | Immunotype and immunohistologic characteristics of tumor-infiltrating immune cells are associated with clinical outcome in metastatic melanoma. | 2012 | Study immune cell infiltration and composition in metastatic melanoma tumours and define three distinct immunotypes. | Immunohistochemistry. | Metastatic melanoma samples (N=183) from N=147 patients. | - Characterized immune cell infiltrates in melanoma metastases, specifically addressing the organization of those cells relative to intratumoral blood vessels, and the prognostic importance of immune cell subsets. - T cells are the dominant infiltrating immune cells, but the second-most prevalent are B lineage cells (B cells and plasma cells), which increase proportionately from Immunotype A to C. |
| Gadeyne | A Multi-Omics Analysis of Metastatic Melanoma Identifies a Germinal Centre-Like Tumor Microenvironment in HLA-DR-Positive Tumor Areas | 2021 | Define the composition and characteristics of the TME of HLA-DR+ vs HLA-DR- metastatic melanoma samples on a single-cell and spatial level. | Multiplexed IHC using the MILAN method, RNA seq and Luminex. | Patient samples including melanoma metastases (N=9) and lymph nodes (N=4). | - B cells and GC B cells were enriched in GCs and TLSs compared to the tumour body. - Neighbourhood analysis found B cells interacting in the GC whereby the various B and plasma cells are interacting with the fDCs cells and T cell zones (with a network of TFH, Th, Tcy and Treg cells). |
| Gorris | Paired primary and metastatic lesions of patients with ipilimumab-treated melanoma: high variation in lymphocyte infiltration and HLA-ABC expression whereas tumor mutational load is similar and correlates with clinical outcome | 2022 | To study primary and metastatic melanoma lesions for tumour mutational burden, HLA status and tumour infiltrating lymphocytes. | Bulk RNA sequencing, immunohistochemistry | Primary and metastatic melanoma lesions from patients (N=33) who received ipilimumab. | - B cell densities showed a poor agreement between the metastatic lesions, and a modest agreement when evaluating invasive margin. - B cells found to be significantly lower in the invasive margin when loss of HLA-ABC was observed. |
| Griss | ReactomeGSA - Efficient Multi-Omics Comparative Pathway Analysis. | 2020 | Introduces ReactomeGSA, a novel tool for multi-omics comparative pathway analysis, to integrate and analyse data from various omics technologies. Analyses the role of B cells in anti-tumour immunity across multiple cancer types, revealing opposing effects of B cells in different cancers. | ReactomeGSA, survival analysis and pathway enrichment. | Single cell RNA sequencing from melanoma tumours (N=31), dataset GSE115978. | - High levels of tumour-induced plasmablast-like cells (TIPB) in melanoma are associated with longer overall survival. - NF-kappaB activation in B cells was significantly up-regulated in melanoma, suggesting a pro-inflammatory role of B cells in anti-tumour immunity. - Reduced NF-KappaB activation and increased NTRK2 signalling were observed in double negative B cells, alongside high IgG expression in double negative and plasma cell populations. |
| Griss | B cells sustain inflammation and predict response to immune checkpoint blockade in human melanoma | 2019 | Study how tumour associated B cells are vital to melanoma associated inflammation. | Induction experiments, FACS analysis, cell viability assays, proteomic analysis, transcriptomic analysis and IHC. | Patients with metastatic melanoma (N=10) were treated with the anti-CD20 antibody ofatumumab. Tumour biopsies were collected (N=9). PBMC samples were taken for EBV-immortalised B cell generation. | - Multiplex immunostaining of whole tissue sections from 41 patients revealed plasmablast-like TAB in 41 samples, plasma cell-like TAB in 37 samples, activated B cell-like TAB in 37 samples, germinal-centre B cell-like TAB in 35 samples, transitional/ Breg-like TAB in 27 samples, and memory B cell-like TAB in 22 samples. - Immortalised patient B cells were cultured with MCM for subsequent RNAseq and proteomic profiling. - TNF signalling via NFKb was one of the most significantly upregulated pathways, known to be associated with inflammation and immune response. |
| Harris | Enriched circulating and tumor-resident TGF-β(+) regulatory B cells in patients with melanoma promote FOXP3(+) Tregs | 2022 | Study B cell phenotype and function by investigating regulatory and pro-inflammatory cytokine-expressing B cells in patients with melanoma. | CyTOF and bulk-RNAseq and scRNAseq. | Melanoma patients (N=26), and healthy volunteers (N=12) for CyTOF analysis. N=103 primary, N=116 metastatic and N=36 metastatic visceral patients for bulk-RNAseq analysis. N=12 treatment naive melanoma patients for scRNA-seq analysis. | - Enrichment in TGF-β and PD-L1-expressing regulatory B cell (Breg) populations and reduced TNF-⍺+ B cell populations in the circulation of patients compared to the healthy volunteer group. - CyTOF analyses of peripheral blood B cells identified enriched circulating TGF-β and PD-L1-expressing CD19+ CD38int IgD+ CD27− Bregs in melanoma patients compared to age- and sex-matched healthy volunteers, suggesting an overall skew toward regulatory cytokine expression in patient B cells. - Bulk RNA-seq analyses of human melanoma samples across primary lesions, metastatic skin, and visceral metastases, confirmed TIL-B (CD79A) gene expression to positively correlate with tumor-infiltrating T lymphocyte (TIL-T) (CD3), and cytotoxic T lymphocyte (CD8A+) gene expression. - Both tumor-infiltrating TGF-β+ (78.6%) and TNF-α+ (87.0%) B cells were predominantly of the class-switched memory phenotype. Analysis indicated that TNF-α expressed by B cells interacted with TNFRII on both Treg and Tcon cells, and TNF-α also signalled via FasR and ICOS expressed by Tregs. |
| Hoch | Multiplexed imaging mass cytometry of the chemokine milieus in melanoma characterizes features of the response to immunotherapy | 2022 | To characterise the chemokine landscape and immune infiltration in metastatic melanoma samples. | Imaging mass cytometry | Patients with advanced (stage III and IV) melanoma (N=69). | - B cells were identified in the tumour samples, as well as densely packed cells indistinguishable between B or T cells (BnT cells). B cell attractant CXCL13 was the most frequently, singly expressed chemokine, predominantly by T cells. - Moderate correlation observed between CXCL13 and B cells and BnT cells, suggesting the importance of CXCL13 for B cell recruitment. - More B cell follicles were found in images classified as low-dysfunctional. B cell follicles were enriched in TCF7+ and TCF7+/PD-1+ T cells. |
| Imahashi | Activated B cells suppress T-cell function through metabolic competition. | 2022 | Investigate the potential contribution of metabolic activity of activated B cells to T cell suppression. | Cell culture, flow cytometry, mass cytometry, bulk and single cell RNAseq. | PBMCs from patients treated with neoadjuvant immune checkpoint blockade (N=6 responders and N=6 non-responders). | - B cells with activated phenotypes can be identified in human tumour tissues and are associated with poor response to ICB therapy. - No difference in B cell clusters between responders and non-responders was observed, except one B cell cluster which accounted for less than 5% of TABs in responders, showed a significant proportional increase in tumour tissues of non-responders (20%) with high expression of B cell activation markers, including CD39, PD-L1 and PD1, suggesting immunosuppressive function. - Also found increased expression of genes related to B cell activation, including CD39, CD80, PD-L1 and PD1 in TABs compared with normal bone marrow B cells. |
| Karagiannis | Innate stimulation of B cells ex vivo enhances antibody secretion and identifies tumour-reactive antibodies from cancer patients. | 2022 | To investigate whether combined stimulation with IL-17, BAFF and CpG supports B cell activation and antibody production *ex vivo* using melanoma patient samples. | Proliferation assay, flow cytometry, ELISA | Melanoma patients (N=3) to study antibody reactivity, melanoma patients to stimulate B cells ex vivo (N=6) | - Significantly higher B cell proliferation when stimulated with IL-17, BAFF and CpG combination compared with CpG alone or IL-17 and BAFF. IL-17+BAFF+CpG stimulation triggered higher IgG titres compared to CpG alone. - Identified tumour reactive antibodies in B cell cultures from patients with melanoma. - Reactivity and expansion of patient B cells to tumour-associated antigens was increased when stimulated with IL-17+BAFF+CpG compared to freshly isolated B cells. |
| Ladányi | Ectopic lymphoid structures in primary cutaneous melanoma. | 2014 | Aimed to study lymphocyte populations in melanoma. | Immunohistochemistry. | Patients with primary cutaneous melanoma (N=147). None of the patients received any antitumor treatment prior to surgery. 76 patients had no metastases developed during the follow-up period, while 21 patients had metastases confined to regional lymph nodes, which were excised. 50 patients developed distant visceral metastases. | - B cells clustered in dense aggregates were observed in 28 of 106 primary melanoma samples (26 %). No differences in size of B-cell aggregates between patients with stage 2 and 3 melanomas. - Cells expressing AID was observed in some cases where B cell aggregates were present. - Analysing the appearance of B cell aggregates with regard to clinicopathologic parameters revealed a higher prevalence in tumours of axial location, especially in those of the head and neck region. - Evaluation of the prognostic relevance of the presence of B cell clusters using Kaplan-Meier analysis yielded no significant association with survival of the melanoma patients. |
| Ladányi | Prognostic impact of B-cell density in cutaneous melanoma. | 2011 | To study the prevalence of B lymphocytes expressing CD20 by immunohistochemistry. | Immunohistochemistry | Primary tumour samples obtained from patients with cutaneous melanoma (N=106) | - Found significant amount of B cells in the peritumoral infiltrate in the majority of samples, while only a small fraction of the cells infiltrated the melanoma cell nests. B cells clustered in dense aggregates resembling follicles were also observed in 28 of 106 samples. - Correlation between the densities of B cells and lymphocytes expressing T-cell activation markers CD25 and especially OX40, as well as their colocalization may suggest a possibility of B cells playing a role in antigen presentation and costimulation of T lymphocytes in primary melanomas. - Kaplan–Meier analysis according to the density of CD20+ lymphocytes revealed that high number of these cells provided significant survival advantage. Low peritumoral B-cell density values combined with low activated T lymphocyte numbers identified a subgroup of patients with strikingly poor survival, while high B-cell/high activated T-cell density groups fared best. |
| Li | The evaluation of tumor microenvironment infiltration and the identification of angiogenesis-related subgroups in skin cutaneous melanoma | 2023 | Study the association between angiogenesis-related genes (ARGs) and tumor microenvironment (TME) infiltration in skin cutaneous melanoma (SKCM). | Gene mutation analysis, clustering, gene set variation analysis (GSVA), deconvolution (CIBERSORT), survival analysis | Patients with skin cutaneous melanoma (N=650). | - Angiogenesis significantly impacts immune infiltration and survival in melanoma. High angiogenic risk is associated with immunosuppressive TME (fewer CD8+ T cells, M1 macrophages, memory B cells). The angiogenesis-related risk model is a useful prognostic biomarker. |
| Song | Role of single cell ferroptosis regulation in intercellular communication and skin cutaneous melanoma progression and immunotherapy | 2023 | To study how ferroptosis, a regulated form of cell death, influences the TME in SKCM, and to assess the potential role of immune cells in prognosis and immunotherapy response. | Bulk RNA-seq, scRNA-seq | ScRNA-seq analysis from melanoma patients (N=31). Bulk RNA-seq data from melanoma tumour samples (N=471). Validation cohorts included normal tissues (N=558) and additional melanoma samples (N=214) | - Significant difference in ferroptosis-regulated B cells abundance was seen in cancer patient compared to normal and ferroptosis-regulated B cell clusters correlate with improved survival in melanoma. |
| Stowman | Lymphoid aggregates in desmoplastic melanoma have features of tertiary lymphoid structures | 2018 | Studying PD-L1 expression in desmoplastic melanoma to identify associations with tertiary lymphoid structures. | Immunohistochemistry | Patients with desmoplastic melanoma (N=23). | - Immune aggregates within desmoplastic melanomas contained organised cell aggregates of CD20+ B cells, surrounded by T cells and dendritic cells. 73% of cases had classical TLS. TLS were evident in the majority of samples where PD-L1 was expressed by melanoma cells. |
| Van de Veen | A novel proangiogenic B cell subset is increased in cancer and chronic inflammation | 2020 | To characterise a novel B cell subset increased in cancer and chronic inflammation. | RNAseq, flow cytometry, bead suspension arrays, qPCR and confocal microscopy. | Advanced metastatic melanoma patients (N=19) and healthy volunteers (N=20). | - Pro-angiogenic IgG4^+^CD73^+^ and VEGF-A^+^CD73^+^ B cells show increased frequencies in circulation and are present in tumour tissue of patients with melanoma compared to healthy volunteers. - Single cell suspensions from metastatic melanoma lesions from 3 patients show that frequency of CD73+ CD49b+ B cells was generally lower among tumour-infiltrating B cells compared to circulating B cells. - IgG4+ B cells were detected in all of the tumour tissues that contained B cells (eight of nine). |
| Werner | A Standardized Analysis of Tertiary Lymphoid Structures in Human Melanoma: Disease Progression- and Tumour Site-Associated Changes with Germinal Centre Alteration. | 2021 | Study multiplex immunostaining of melanoma tissues to characterise TLS phenotypes, disease progression and site-associated changes in TLS phenotype, density and spatial distribution. | Seven colour multiplex immunohistochemical staining | Patients with primary cutaneous melanoma (N=48). N=27 patients presented without metastasis within a follow-up interval of up to 140 months. N=21 patients were diagnosed with regional metastasis at the time of first diagnosis. | - Identified TLS in 16 of the 48 primary tumours, predominantly with early TLS phenotype. Only 4 tumour samples presented with a secondary follicular TLS and one of these had BCL6+ lymphatic cells. In samples with a higher Breslow thickness and the presence of TLS, early TLS appeared at a higher density within the 1 mm perimeter of the intratumoural compartment. - Extratumoral TLS were mostly present within the 1 mm perimeter with a drop in density with the 2 to 6 mm perimeters. - Alternatively, TLS was found in 45 of 55 metastatic melanoma samples. Early TLS were the most prevalent TLS phenotype, followed by secondary follicular TLS with a BCL6 -germinal centre and only small proportions of both secondary follicular TLS with a BCL6 + germinal centre and primary follicular TLS. - TLS density and relative area was also increased in metastatic vs primary tumours. |
| Werner | Loss of Lymphotoxin Alpha-Expressing Memory B Cells Correlates with Metastasis of Human Primary Melanoma. | 2021 | To study expression of lymphotoxin alpha and interleukin-10 in different antigen-experienced B cell populations. | Immunohistochemistry | Melanoma patient samples (N=59), Caucasian. | - LTA and IL-10 expression was not restricted to a distinct B cell subtype but detected in activated and memory-like B cells and antibody secreted cells. Expressing-cells were found predominantly at the invasive tumour-stoma front. - Primary melanoma samples with metastasis had significantly fewer LTA+ memory B cells and a trend in reduced LTA+ activated B cells compared to non-metastatic patients. A significant association between LTA+ memory B cells and Breslow depth was observed. - Enrichment of IL-10+ activated B cells was found at lymph node sites compared to metastatic skin sites. |
| Wu | PD-L1(+) regulatory B cells act as a T cell suppressor in a PD-L1-dependent manner in melanoma patients with bone metastasis. | 2020 | Examine the expression of PD-L1 by B cells, and investigate the regulatory functions mediated by PD-L1+ B cells and study the association between PD-L1+ B cells and bone metastasis in melanoma patients. | FACS, IFN-γ suppression assay | Melanoma patients (N=59), including 34 male patients and 25 female patients between 44 and 70 years of age, and healthy individuals (N=20), including 12 male subjects and 8 female subjects between 45 and 70 years of age. Staging was performed in accordance with the TNM system, in which all the Stage IV patients were with bone metastasis. | - In melanoma patients, the frequency of PD-L1+ B cells significantly increased to 2.1 % in stage I, 2.6 % in stage II, 4.9 % in stage III, and 8.5 % in stage IV, however large variations in the frequency of PD-L1+ B cells were observed among the melanoma patients, especially in stage IV patients. - Compared to total B cells, the PD-L1+ B cells presented significantly higher IgM and IgD, which were expressed by mature naive and inactivated B cells. - Expression of cytokines including IL-10, TGF-β, and IL-35 subunits IL-12A and EBI3 was lower in PD-L1+ B cells than in total B cells. - A subset of B cells presented high expression of PD-L1 and possessed the capacity to inhibit T cell IFN-γ expression in a PD-L1-dependent manner. |
| Yan | Understanding heterogeneous tumour microenvironment in metastatic melanoma | 2019 | Investigating immune infiltration in metastatic melanoma, focusing on the spatial distribution and interaction of immune cells with the tumour microenvironment. | Multiplexed immunofluorescence (MxIF) imaging, cell DIVE technology, spatial analysis algorithms (CAA and CNAA) | Lymph node biopsies from metastatic melanoma patients (N=4) for Cell DIVE, lymph node metastases from stage III melanoma patients (N=158). | - CD20+ B cells were observed in the TME. CD20+ B cells infiltration is correlated with T cells. |
| Yu | A Pan-Cancer Analysis of Tumour-Infiltrating B Cell Repertoires | 2022 | Study extracted BCR repertoires from tumour and adjacent non-tumour samples in the cancer genome atlas. | Bulk RNAseq | Primary melanoma tumours (N=103), metastatic tumours (N=365) and normal adjacent tissue (N=1). | - Pielou's evenness index for each chain type, which reflects the evenness of the clone distributions within each sample, shows that SKCM has the lowest clone evenness (distribution) compared to other tumour types. - Network analysis to reveal differences in clonal expansion and diversification across tumour types and between tumour/non tumour samples demonstrated that SKCM had one of the highest mean vertex Gini indexes across the Ig chains, indicating higher levels of clonal expansion in these tumour types. This suggests that few clones dominate the humoral repertoire. - A higher IGH evenness was associated with decreased survival in SCKM, suggesting that B cells may play different roles in this tumour compared to others (where evenness associated with better OS). |

Supplementary Table 10. Studies investigating B cell responses in melanoma patients in the context of Prognosis and Survival

| Author | Title | Year | Aims of Study | Techniques Used | Patient Demographics | Key Findings | Association between B cells and survival in melanoma |
| --- | --- | --- | --- | --- | --- | --- | --- |
| Andrés | Prognostic value of quantitative immune alterations in melanoma patients | 2005 | To assess quantitative immune alterations in melanoma patients, comparing disease-free individuals with those having metastatic disease, and to evaluate the prognostic value of these immune changes. | Flow cytometry Nephelometric methods | Melanoma patients (N=86): disease-free (N=63) and with distant metastases (N=23) | - B lymphocyte counts were significantly lower in metastatic patients compared to disease-free patients. - Immunoglobulin A levels are significantly higher in patients with active disease. | No association (no survival analyses conducted) |
| Attrill | Detailed spatial immunophenotyping of primary melanomas reveals immune cell subpopulations associated with patient outcome. | 2022 | To characterize the tumour immune microenvironment (TIME) of primary melanomas, focusing on immune cell subpopulations, particularly CD8+ T cells and B cells, to identify biomarkers associated with patient outcomes and recurrence-free survival | Flow cytometry Multiplex immunohistochemistry  Machine learning models | Patients with primary melanoma (N=66), stage II (N=39) and stage III (N=27) | - Higher intratumoral B cell density was associated with improved recurrence-free survival (RFS). - B cells localized closer to melanoma cells in patients with good outcomes. - Increased B cell presence at the tumor/stromal interface correlated with better prognosis. | **Positive association** |
| Bosisio | Plasma cells in primary melanoma. Prognostic significance and possible role of IgA | 2016 | To investigate the prognostic significance of plasma cells (PCs) in primary cutaneous melanoma and explore the role of IgA produced by these PCs in melanoma progression. | Immunohistochemistry  Gene Scan Analysis | Primary cutaneous melanomas (N=710); Melanoma subtypes included superficial spreading (SSM), nodular (NM), acral lentiginous (ALM), and others | - PCs were found in the inflammatory infiltrate of 3.7% of the melanoma samples, reporting higher prevalence in thicker, ulcerated, and mitotically active tumours. - PC-rich melanomas (clusters/sheets of PCs) were associated with worse survival, while sparse PCs correlated with better outcomes. - PCs were polyclonal, predominantly expressing IgG and IgA, with IgA showing oligoclonality, suggesting an antigen-driven response. - IgA+ PCs were more abundant in PC-rich melanomas and their draining lymph nodes, indicating a systemic immune response. - Tertiary lymphoid structures (TLS) were rare in primary melanomas, suggesting PCs likely originated elsewhere (e.g., lymph nodes). | **Negative association** |
| Brase | Role of Tumor-Infiltrating B Cells in Clinical Outcome of Patients with Melanoma Treated with Dabrafenib Plus Trametinib. | 2021 | To investigate the role of B cells as potential biomarkers in predicting clinical outcomes for BRAF V600-mutant metastatic melanoma patients treated with dabrafenib plus trametinib (a BRAF/MEK inhibitor combination). It explores how baseline B-cell gene signatures and infiltration correlate with treatment response and survival outcomes. | Gene expression profiling (GEP)  CD3/CD19 IHC staining  Kaplan-Meier survival analysis Cox regression modelling  Spatial proximity analysis | Patients with melanoma (N=146); all had BRAF V600-mutant metastatic melanoma. BRAF mutation: V600E (91%), V600K (8%); metastasis stage: M0 (5%), M1 (95%). | - High baseline B-cell infiltration was associated with decreased survival in patients treated with dabrafenib plus trametinib, possibly due to immunosuppressive features of B-cell-rich tumours. - High T cell/low B cell signatures correlated with prolonged survival compared to high T cell/high B cell signatures. - High baseline B-cell infiltration correlated with decreased MAPK pathway activity, suggesting a modified tumour phenotype. B cells in the tumour compartment were in close spatial proximity to T cells. - B-cell-rich tumours exhibited immunosuppressive features, including increased HLA-DR/IDO-1 expression, which correlated with poor prognosis. | **Negative association** |
| Cabrita | Tertiary lymphoid structures improve immunotherapy and survival in melanoma. | 2020 | The study focuses on the role of tertiary lymphoid structures (TLSs) in the tumour immune microenvironment of melanoma, particularly their impact on immunotherapy response and patient survival. It investigates the interplay between B cells and T cells in melanoma tumours and how TLSs influence the immune response to checkpoint blockade therapies. | Immunohistochemistry (IHC) Immunofluorescence RNA sequencing and microarray data Single-Cell RNA Sequencing (scRNA-seq) Digital Spatial Profiling | Patients with melanoma (N=177) Stage: N=104 patients had regional metastatic disease (stage III), N=50 had distant disease (stage IV), and N=19 had local disease (stage II). Additional Cohorts: The study also included smaller cohorts of patients treated with anti-CTLA4 (N=37) and anti-PD1 (N=40) therapies. | - 25% of melanoma tumours contained CD20+ B cell clusters. CD20+ B cell clusters were surrounded by mainly CD4+ T cells, indicating the formation of TLS. - High expression of CD20 in melanoma tumours was associated with improved patient survival. - Patients with both CD20+ B cells and CD8+ T cells had the best survival outcomes, while those without either had the worst outcomes. In multivariate survival analysis, the combination of CD20+ B cells and CD8+ T cells was significantly associated with improved survival. - High TLS gene signature predicted better overall survival in metastatic melanoma. - Patients with high TLS expression had improved response to anti-CTLA-4 and anti-PD-1 therapy. | **Positive association** |
| Damsky | B cell depletion or absence does not impede anti-tumour activity of PD-1 inhibitors. | 2019 | The study aimed to assess the role of B cells in the anti-tumour activity of PD-1 inhibitors in melanoma. | Immunohistochemistry, flow cytometry | Melanoma patients treated with anti-PD-1 monotherapy (N=40). Majority of patients had cutaneous melanoma, with mutations in BRAF (30%), NRAS (22.5%), KIT (5%), and GNAQ (2.5%). | - B cells were sparse in pre-treatment melanoma tumours, and their presence did not correlate with response to anti-PD-1 or overall survival. | No association |
| Freeman | Combined tumour and immune signals from genomes or transcriptomes predict outcomes of checkpoint inhibition in melanoma. | 2022 | The study aimed to identify combinations of tumour and immune signals from genomic or transcriptomic data that predict outcomes of checkpoint inhibition (CPB) in melanoma, focusing on improving prediction models using DNA or RNA assays. | Whole exome sequencing  bulk RNA sequencing  Rearranged TCR/Ig sequences. | Primary cohort: melanoma patients with WES data (N=189) and with RNA-seq data (N=178). Secondary cohort: melanoma patients from independent datasets treated with PD-1 or combination CTLA-4/PD-1 (N=180). | - B cell burden (BCB) derived from rearranged Ig sequences in DNA or RNA correlated with immune infiltration and predicted improved outcomes when combined with high tumour mutational burden (TMB). - Patients with high BCB and high TMB had longer overall survival (OS) and higher response rates to ICI. BCB levels were higher in RNA than in DNA, likely due to high Ig expression in plasma B cells. - BCB dynamics showed that B cell infiltration did not significantly increase post-treatment, unlike T cells. | **Positive association** |
| Garcia-Mulero | Additive role of immune system infiltration and angiogenesis in uveal melanoma progression | 2021 | The study aimed to investigate the role of immune system infiltration and angiogenesis in uveal melanoma (UM) progression, focusing on their additive effects on prognosis and metastatic potential. | RNAseq | Primary uveal melanoma patients (N=213) from 5 datasets (TCGA and GEO repositories) | - B-cell infiltration was consistently associated with better prognosis across multiple analysis methods, while most other immune cells (e.g., CD8+ T cells, NK cells, macrophages) and stromal cells correlated with poor prognosis. - High combined scores for angiogenesis and antigen presentation identified a subgroup with aggressive UM and metabolic pathway activation. | **Positive association** |
| Garg | Tumour-associated B cells in cutaneous primary melanoma and improved clinical outcome | 2016 | The study investigates the role of tumour-Associated B cells (TAB) in cutaneous primary melanoma, focusing on their association with patient prognosis and overall survival. It explores whether the presence and number of TAB in melanoma tumours can serve as a prognostic biomarker. | Immunohistochemistry (IHC) Quantitative Digital Imaging RNA Expression Analysis | Cohort 1: patients with primary cutaneous melanoma (N=57), aged 31-89 years. No metastasis (N=43), and metastasis at diagnosis (N=14). Cohort 2: Patients with primary cutaneous melanoma (N=41), aged 25-78 years. N=16 patients developed metastasis, and N=25 did not. Cohort 3: Cutaneous melanoma samples from TCGA (N=345) with mRNA expression and survival data. | - Primary melanomas without metastasis had significantly higher numbers of TAB compared to those that metastasized. - Higher numbers of TAB were associated with significantly better overall survival in patients with primary melanomas >1 mm Breslow depth. - Patients with CD20+-high tumours had a significantly longer overall survival than patients with CD20+-low tumours, particularly when presenting with nonulcerated tumours. - In TCGA dataset, patients with high CD19/CD20 expression had significantly better survival. | **Positive association** |
| Hillen | Leukocyte infiltration and tumour cell plasticity are parameters of aggressiveness in primary cutaneous melanoma | 2007 | The study aimed to investigate the prognostic value of leukocyte infiltration and tumour cell plasticity in primary cutaneous melanoma, focusing on the relationship between immune cell subsets, tumour aggressiveness, and patient survival. | Immunohistochemistry | Patients with melanoma (N=58); 64% superficial spreading melanoma, 36% nodular melanoma | - B cells (CD20+) were more abundant in nodular melanomas (aggressive subtype) compared to superficial spreading melanomas. - Higher peritumoral B cell infiltration correlated with advanced tumour stages (e.g., Breslow depth >3 mm). - CD20+ expression was not significantly associated with overall survival. | No association |
| Huang | A BRAF mutation-associated gene risk model for predicting the prognosis of melanoma | 2023 | The study aimed to explore BRAF mutation-related biological features, constructing a prognostic signature using key genes, and investigating its association with immune cell infiltration and immune checkpoint molecules to predict melanoma prognosis. | Gene Set Enrichment Analysis (GSEA) Univariate and LASSO-Cox Regression Risk Score Model Immune Infiltration Analysis with CIBERSORT Nomogram Construction | RNA sequencing and clinical data from melanoma patients (N=467) in the TCGA database (N=233 with BRAF mutations, 54.3%) and validation data from the ICGC cohort. | - The study found significantly higher proportions of naïve B cells and plasma cells in the low-risk group, which also showed higher expression of immune checkpoint molecules (PD-1, CTLA4, CD80/CD86). - On the other hand, high-risk patients had fewer B cells but more immunosuppressive macrophages (M0/M2), highlighting an inverse relationship between B cell presence and immune evasion in melanoma progression. | **Positive association** |
| Iglesia | Genomic Analysis of Immune Cell Infiltrates Across 11 Tumour Types. | 2016 | To analyse the prevalence, prognostic relevance, and clonal diversity of immune cell infiltrates, particularly B cells, across 11 tumour types using genomic data to understand their association with survival and potential as biomarkers for immunotherapy response. | Single-cell RNA sequencing | Melanoma samples (N=329). | - BCR diversity was associated with survival, with low diversity linked to improved outcomes in melanoma. | **Positive association** |
| Kang | Significance of Tumour Mutation Burden in Immune Infiltration and Prognosis in Cutaneous Melanoma | 2020 | The study investigates the significance of tumour mutation burden (TMB) in immune infiltration and prognosis in cutaneous melanoma. It explores the relationship between TMB, immune cell infiltration, and gene expression profiles to predict melanoma prognosis and response to immunotherapy. | RNA-seq | Cutaneous melanoma patients (N=449) from the TCGA database with complete somatic mutation, transcriptome, and clinical data. Pathological Stage: 37.42% stage III, 29.62% stage II, 18.93% stage I, 4.90% stage IV. | - Lower TMB is associated with higher levels of memory B cells. - High infiltration level of B cell was associated with better survival outcomes. - However, cox regression analysis shows no significant association with prognosis in melanoma patients. | **Positive association** |
| Lardone | Cross-platform comparison of independent datasets identifies an immune signature associated with improved survival in metastatic melanoma. | 2016 | The study aimed to identify a common gene expression signature associated with improved survival in metastatic melanoma patients by comparing independent datasets using a threshold-free algorithm, and to explore the role of immune cells, particularly B cells, in favourable outcomes. | RNAseq, Immunohistochemistry | Datasets included Stage III and IV metastatic melanoma patients from publicly available studies GSE22153 (n=57 patients), GSE46517 (n=25 primary cutaneous and 61 metastatic melanoma specimens), and GSE19234 (n= 44 metastatic melanoma tissue samples from 38 patients). | - B cell-associated genes were significantly enriched in tumours from favourable outcome patients. - Immunohistochemistry confirmed higher levels of CD20+ B cells in close proximity to T cells in favourable outcome tumours compared to poor outcome tumours. - The favourable outcome signature included genes involved in B cell-T cell interactions, such as MHC class II molecules and costimulatory proteins. | **Positive association** |
| Lundberg | B cell-related gene signature and cancer immunotherapy response. | 2021 | The study aimed to identify a B cell-related gene (BCR) signature that influences the prognostic effects of tumour-infiltrating B cells and predicts response to cancer immunotherapy. | Gene expression analysis with clinical follow-up information | Melanoma patients (N=325). Anti-PD1-treated (N=121) and anti-CTLA4-treated (N=40) melanoma patients. Pre-treatment gene expression profiles available. | - Higher B cell abundance was associated with favourable survival only when the B cell-related gene signature (comprising 9 cytokine signalling genes) was low. - The B cell-related signature predicted ICB response in melanoma, outperforming established markers. | **Positive association** |
| Lynch | Heterogeneity in tertiary lymphoid structure B-cells correlates with patient survival in metastatic melanoma | 2021 | The study aimed to investigate the prognostic significance of tertiary lymphoid structures (TLS) in metastatic melanoma, focusing on their association with patient survival, immune cell infiltration, and the functional heterogeneity of B cells within TLS. | Multiplex immunofluorescence histology  Tissue microarray | Patients with stage IIIB–IV cutaneous melanoma metastases (N=64), all checkpoint-blockade naïve. | - TLS+ tumours exhibited higher densities of intratumoral B cells and plasma cells compared to TLS-negative tumours. Intra-TLS B cells showed significant heterogeneity in activation markers, with higher fractions of AID+ B cells (undergoing somatic hypermutation) and CD21+CD20+ B cells correlating with improved overall survival (OS). | **Positive association** |
| Martinez-Escribano | Changes in the number of CD80+, CD86+, and CD28+ peripheral blood lymphocytes have prognostic value in melanoma patients | 2003 | To investigate whether the concentration of peripheral blood lymphocytes expressing costimulatory molecules (CD80, CD86, CD28) could serve as prognostic markers in melanoma patients, correlating with tumour thickness and survival outcomes. | Flow cytometry | Patients with primary cutaneous melanoma (N=38) and healthy controls (N=27) | - CD19+CD80+ B cells were significantly higher in patients with thinner tumours (≤2 mm) and those surviving 3 years, suggesting a protective role. - CD19+CD80+CD86+ B cells also increased in patients with better prognosis, though less markedly than CD80+ alone. | **Positive association** |
| Martinez-Rodriguez | A significant percentage of CD20-positive TILs correlates with poor prognosis in patients with primary cutaneous malignant melanoma. | 2014 | This study aimed to study how tumour-infiltrating B lymphocytes influence disease progression in melanoma patients, and how they could be potentially used as a prognostic marker. | Immunohistochemistry | Primary cutaneous melanoma patients (N=91). 51 women and 40 men, with ages between 21-87 years. | - Patients with a significant population (>15%) of CD20+ tumour-infiltrating lymphocytes were found to be associated with worse prognosis; specifically, the development of tumour recurrence, a shorter disease-free interval, the development of lymph node metastasis, and shorter overall survival. | **Negative association** |
| Schina | Intratumoral T-cell and B-cell receptor architecture associates with distinct immune tumour microenvironment features and clinical outcomes of anti-PD-1/L1 immunotherapy | 2023 | The study aimed to investigate how the diversity of intratumoral T-cell and B-cell receptor (TCR/BCR) repertoires influences the tumour immune microenvironment (TIME) and clinical outcomes in patients treated with anti-PD-1/PD-L1 immunotherapy. | TCR and BCR, bulk-RNA-Seq Machine learning models | Data sourced from The Cancer Genome Atlas (TCGA; 30 cancer types) and 10 additional immunotherapy datasets. Melanoma data is from pre-treatment advanced melanoma patients receiving anti-PD1 (n = 268). | - BCR diversity was strongly associated with antibody production pathways but not with overall B-cell infiltration or CD8+ T-cell activity. - High BCR richness correlate with improved survival in melanoma (SKCM). | **Positive association** |
| Selitsky | Prognostic value of B cells in cutaneous melanoma. | 2019 | The study aimed to investigate the prognostic and predictive value of B cells and their receptor (BCR) repertoires in cutaneous melanoma (SKCM), using RNA sequencing data to understand their association with clinical outcomes and response to immunotherapy. | RNA sequencing  BCR repertoire assembly using the bioinformatics tool V’DJer. TCR repertoire analysis using MiXCR. | SKCM patients from TCGA (N=473) (GDAC Firehose from June of 2016) | - Clonally restricted BCR repertoires were favourably prognostic in SKCM. - Higher B cell diversity was observed in female patients compared to males. - BCR diversity and abundance were not correlated with tumour mutation burden, suggesting B cells target non-mutated antigens. - Lack of assembled BCR in pre-treatment tumours was associated with poor response to CTLA-4 inhibitors. - Memory B cell signatures were linked to improved survival, while regulatory B cell signatures were associated with worse outcomes. | **Positive association** |
| Therien | Spatial biology analysis reveals B cell follicles in secondary lymphoid structures may regulate anti-tumour responses at initial melanoma diagnosis. | 2022 | The role of B cell follicles in secondary lymphoid structures, particularly in sentinel lymph nodes (SLNs), and their potential regulation of anti-tumour responses in melanoma patients at the time of initial diagnosis. The research aims to understand how B cell activation and spatial arrangement within SLNs may influence tumour progression and patient outcomes. | Flow Cytometry NanoString Digital Spatial Profiling (DSP) | Flow Cytometry Cohort: melanoma patients (N=13) (N=3 with tumour in SLN, N=10 without). DSP Cohort: melanoma patients (N=24) (N=8 with tumour in SLN, N=16 without). | - B cell follicles in SLNs with tumour (pSLN) showed higher expression of activation markers (e.g., Ki-67, CD40, HLA-DR) compared to SLNs without tumour (nSLN). - Patients with B cells infiltrating the tumour had prolonged recurrence-free survival (10 years) compared to those with B cells excluded from the tumour. - Patients with infiltrative B cell patterns had higher expression of CD8, CD45, and GZMB, while those with excluded patterns had higher expression of immunosuppressive markers like B7-H3 and ICOS. | **Positive association** |
| Versluis | Interferon-gamma signature as prognostic and predictive marker in macroscopic stage III melanoma. | 2024 | The study aimed to identify prognostic and predictive biomarkers, specifically interferon-gamma (IFNγ) and B cell signatures, to improve adjuvant treatment selection for patients with macroscopic stage III melanoma. | RNA sequencing  Immunohistochemistry | Patients with stage III melanoma (N=98), (N=49 observation, N=49 adjuvant intention) | - B cell score was prognostic in untreated patients, with high B cell scores associated with longer recurrence-free survival (RFS). - B cell score did not show predictive value for adjuvant therapy benefit. - B cell presence correlated with CD20+ staining in tumour regions but was not independent of IFNγ score. | **Positive association** |
| Wang | Characterization of the treatment-naive immune microenvironment in melanoma with BRAF mutation | 2022 | The study aimed to characterize the immune microenvironment in treatment-naive melanoma with BRAF mutations compared to BRAF wild-type (BRAF-wt) melanoma to understand differences in immune composition and their implications for immunotherapy response. | Single-cell RNA sequencing Bulk RNA sequencing Flow cytometry Multiplex immunohistochemistry  Data deconvolution | A total number of 4645 cells from melanoma patients (N=19): including patients wt for NRAS and BRAF (N=8), patients with NRAS mutation (N=5), with BRAF V600 mutations (N=4) (BRAF-V600E=3, BRAF-V600K=1) and patients with unknown mutation status (N=2) | - BRAF-mutant melanoma showed significantly increased B cell infiltration compared to BRAF-wt tumours. - B cells were associated with a trend toward improved survival in BRAF-mutant patients. - No significant survival association was observed for B cells in BRAF-wt patients. | **Positive association** |
| Xiong | Prognostic value of the expression of chemokines and their receptors in regional lymph nodes of melanoma patients | 2019 | The study aimed to investigate the prognostic value of chemokines and their receptors in regional lymph nodes (LNs) of melanoma patients, focusing on their relationship with immune cell infiltration and patient survival. | RNAseq | Melanoma patients (N=221); 42.53% Stage 0/I/II, 46.61% Stage III/IV. Including in situ SKMC tissue (N=103), distant metastatic tissue (N=63), adjacent tissue (N=74) and not identified tissue sources (N=3). | - High expression of CCR4, CCR6-9, CCL13, CCL22, CCL23, and XCR1 was positively correlated with memory B cells and naive T cells. These chemokines/receptors were negatively correlated with M0 macrophages and resting mast cells, suggesting a role in promoting anti-tumour immunity. - The survival prognosis of the patients with high expression of these chemokines and receptors was found to be better than in the low expression group. | **Positive association** |
| Yan | Prognostic Role of Tumour Mutation Burden Combined with Immune Infiltrates in Skin Cutaneous Melanoma Based on Multi-Omics Analysis | 2020 | The study aimed to investigate the prognostic role of tumour mutation burden (TMB) combined with immune infiltrates in skin cutaneous melanoma (SKCM), focusing on the relationship between TMB, immune cell infiltration, and patient survival outcomes. | bulk RNA-seq | SKCM patients from TCGA (N=454) Staging: 49.78% stage I/II, 42.29% stage III/IV, 7.93% unknown | - Lower infiltration levels of B cells were associated with poor survival outcomes in SKCM. - Diverse forms of copy number variations (CNVs) in hub immune genes (e.g., CNTFR, CRABP2) correlated with reduced B cell infiltration in the tumour microenvironment. | **Positive association** |
| Zhao | A multi-omics deep learning model for hypoxia phenotype to predict tumour aggressiveness and prognosis in uveal melanoma for rationalized hypoxia-targeted therapy | 2022 | The study aimed to investigate the role of hypoxia in uveal melanoma (UM) by analysing its association with tumor aggressiveness, prognosis, and the tumor microenvironment, with the goal of improving hypoxia-targeted therapy. | Multi-omics analysis: mRNA, lncRNA, miRNA, DNA methylation, SNV, CNV Deep neural network: DNN2HM analysis (CIBERSORT, ESTIMATE). Functional enrichment analysis: GO, KEGG. | Primary cohort: uveal melanoma patients from TCGA (N=80). Validation cohorts: UM patients from GSE22138 (N=63) and UM patients from GSE84976 (N=28). | - Hypoxic UM tumours exhibited higher genomic instability, increased CD8+ T cell infiltration, and decreased naïve B cell levels. - Reduced naïve B cell levels were associated with poorer prognosis, highlighting their protective role in UM. | **Positive association** |
| Zhou | The prognostic implications of cuproptosis-related gene signature and the potential of PPIC as a promising biomarker in cutaneous melanoma | 2024 | To investigate the role of cuproptosis-related genes (CRGs) in melanoma prognosis, immune microenvironment and therapy resistance. A cuproptosis-related gene signature (CGS) was developed to predict patient survival and immune cell infiltration patterns. | RNAseq | Normal skin tissue samples (N=812) (GTEx database). Melanoma patients (N=451) (TCGA dataset). Patients analysed for gene mutations (N=469). | - Cuproptosis-­related gene signature was negatively correlated with B cells. - Increased B cells level led to longer overall survival. - B cells level was significantly higher in low-risk group. | **Positive association** |

Supplementary Table 11. Studies investigating B cell responses in melanoma patients in the context of response to treatment

| Author | Title | Year | Aims of Study | Techniques Used | Patient Demographics | Key Findings | Association between B cells and response to treatment |
| --- | --- | --- | --- | --- | --- | --- | --- |
| Aklilu | Depletion of normal B cells with rituximab as an adjunct to IL-2 therapy for renal cell carcinoma and melanoma | 2004 | To assess B cell depletion in melanoma patients receiving rituximab as an adjunct to IL2 therapy. | Flow Cytometry | Metastatic melanoma patients (N=6) | - B cell depletion was achieved after four weekly doses of rituximab, and serum immunoglobulin levels were not affected by drug administration. - *Ex vivo* expansion of T cells before and after rituximab showed no difference in cytokine production, indicating no benefit of B cell depletion with rituximab as an adjunct to IL2 therapy. | No association |
| Anagnostou | Integrative Tumour and Immune Cell Multi-omic Analyses Predict Response to Immune Checkpoint Blockade in Melanoma | 2020 | Analyses of genomic, transcriptomic and immune cell signatures to study the interplay between cancer and immune cells during checkpoint inhibitor immunotherapy. | WES, RNA sequencing, TCR sequencing | Metastatic melanoma patients treated with anti-PD1, anti-CTLA4 or combination (N=64). | - Increased Ig gene rearrangement and tumour-associated B cells in baseline tumours from responders compared to non-responders. - The abundance of both naïve B cell and plasma cells at baseline were significantly higher in responders. - Increased Ig gene rearrangements were also associated with improved progression-free survival. Clonal counts for both IgH and IgL chains were significantly increased in responding patients. | **Positive association** |
| Das | Early B cell changes predict autoimmunity following combination immune checkpoint blockade | 2017 | Analysing B cell changes in melanoma patients undergoing treatment with either anti-CTLA4 or anti-PD1, or in combination. | Flow Cytometry | Patients with advanced melanoma receiving immune checkpoint blockade, including combination (N=23), anti-CTLA4 (N=8) and anti-PD1 (N=8) monotherapy | - Circulating B cells decreased in patients following combination ICB, but not in patients treated with anti-PD1/anti-CTLA4 monotherapy. - However specific populations were increased on treatment, including class switched memory B cells and plasmablasts in the combination cohort and CD21lo B cells in the combination and anti-CTLA4 cohorts. CD21lo IgD- CD27+ B cells were identified as a specific target of combination therapy, as it was the only proliferating subset post-treatment. - An early decline in B cell numbers after therapy directly correlated with onset of toxicity and associated with worse overall survival in patients with high grade toxicity. | **Positive association** |
| Ding | Antigen presentation by clonally diverse CXCR5+ B cells to CD4 and CD8 T cells is associated with durable response to immune checkpoint inhibitors | 2023 | Utilised transcriptional analysis and patient clinical outcomes to investigate immune mechanisms driving tumour response to immune checkpoint and MAPKi therapies. | RNA sequencing | Tumour samples from melanoma patients (N=48) treated with ICI (anti-PD1) or MAPK inhibitors | - Increased B cell abundance was found in on-treatment responding patients receiving ICI compared to MAPKi and compared to non-responding patients. B cell and TLS signatures were associated with improved overall survival after ICI but not MAPKi therapy. - Patients with ICI-responding tumours promoted improved receptor-ligand interaction with T helper cells via MHC II pathway. - Higher BCR diversity was associated with improved survival after ICI but not MAPKi therapy, and BCR clonality was not associated with survival in either cohort. | **Positive association** |
| Dollinger | Divergent resistance mechanisms to immunotherapy explain responses in different skin cancers | 2020 | The study aimed to investigate divergent resistance mechanisms to immunotherapy in melanoma by analysing immune cell interactions, particularly focusing on memory B cells and macrophages, and to develop a mathematical model predicting optimal immune cell ratios for treatment response. | Single-cell RNA sequencing | CD45+ sorted cells from patients with metastatic melanoma (N=32), treated with anti-PD-1, anti-CTLA-4, or combination therapy. | - Memory B cells were significantly more abundant in immunotherapy responders compared to non-responders in melanoma. - Memory B cells in responders exhibited higher activation scores post-treatment, while non-responders showed increased anergy. - Inhibitory signalling from macrophages to memory B cells (via FCGR2B pathway) was stronger in melanoma non-responders, suppressing B cell activity. | **Positive Association** |
| Egan | Small Gene Networks Delineate Immune Cell States and Characterize Immunotherapy Response in Melanoma | 2023 | To understand how immune cell states within the tumour microenvironment influence response to checkpoint inhibitor therapy, with the aim of identifying biomarkers for prediction of response. | Single cell and bulk RNA sequencing | Discovery Dataset: metastatic melanoma patients (N=19) treated with ICI. Validation Dataset: patients from four independent studies (N=209), including advanced melanoma patients treated with anti-PD-1, anti-CTLA-4, or combination therapy. | - B cells were found to be associated with responders, reporting enrichment of IFN-gamma and TNF-alpha pathway activity in these patients. | **Positive association** |
| Gatto | Early Increase of Circulating Transitional B Cells and Autoantibodies to Joint-Related Proteins in Patients with Metastatic Melanoma Developing Checkpoint Inhibitor-Induced Inflammatory Arthritis | 2023 | To investigate associations between B cell-related changes and development of inflammatory arthritis after treatment with immune checkpoint inhibitors. | Flow Cytometry, autoantibody detection | Metastatic melanoma patients treated with immune checkpoint inhibitors with no irAEs (N=15) and inflammatory arthritis (IA) (N=7) | - The proportion of circulating B cells was higher in patients with IA compared to no irAEs after ICI. - During treatment circulating B cells declined in the non-irAEs patients, whilst in the IA patients no significant change in circulating B cells was observed. - An enrichment in the proportion of transitional B cells was associated with development of IA after ICI therapy. - Patients that developed IA were significantly younger at the time of melanoma diagnosis compared to non-irAEs patients. - A significant increase in autoantibody production to type II collagen epitopes was detected in patients that developed IA compared to those who did not develop irAEs. | **Negative association** |
| Helmink | B cells and tertiary lymphoid structures promote immunotherapy response | 2020 | The study aimed to investigate the role of B cells and tertiary lymphoid structures (TLSs) in promoting responses to immune checkpoint blockade (ICB) therapy in melanoma patients. | Bulk and single cell RNA sequencing, Mass cytometry, Immunohistochemistry (IHC) and multiplex IF, Digital Spatial Profiling,  BCR clonotype analysis, Microenvironment Cell Populations-counter (MCP-counter) for immune cell deconvolution | Melanoma patients on a clinical trial of neoadjuvant ICB (N=23). Patients received nivolumab (N=12) or ipilimumab (N=11). Validation melanoma cohort used patients enrolled in OpACIN neo trial (N=18), stage III patients. Targeted therapy cohort of patients received DAB-TRAM for BRAF-mutated melanoma (N=13). | - B cells and TLSs were enriched in tumours of ICB responders, with memory B cells and plasma cells showing clonal expansion and functional activation, suggesting their critical role in enhancing anti-tumour immunity. - Switched memory B cells (CXCR3+, CD86+) are key, potentially aiding antigen presentation and cytokine production. - B cell signatures predict better survival and treatment response, independent of T cell infiltration. | **Positive association** |
| Liu | Identification of subtypes correlated with tumour immunity and immunotherapy in cutaneous melanoma | 2021 | Identification of immune subtypes of melanoma using transcriptome datasets from six melanoma patient cohorts from both treatment naïve patients and patients receiving checkpoint inhibitors. | Single cell RNA sequencing | TCGA-melanoma dataset (N=472), GSE65904 (N=214), GSE98394 (N=78), GSE53118 (N=79), ICI-R-melanoma (N=182), ICI-M-melanoma (N=65), GSE72056 4 (485 single cells) | - Three immune subtypes were determined (Im-H, Im-M and Im-L), representing high, medium and low immunity subtypes, respectively. - In the TCGA melanoma cohort, Im-H displayed significantly prolonged overall survival than Im-M, and Im-M showed better overall survival than Im-L. - B cell receptor signalling was significantly upregulated in responding patients versus non-responders. | **Positive association** |
| Mastracci | Response to ipilimumab therapy in metastatic melanoma patients: potential relevance of CTLA-4+ tumour infiltrating lymphocytes and their in situ localization | 2020 | Investigating how the density, type and localisation of tumour-infiltrating lymphocytes correlates to response to treatment with ipilimumab in metastatic melanoma patients, aiming to identify predictive biomarkers for immunotherapy response. | Haematoxylin and eosin (H&E) staining and immunohistochemistry (IHC) | Metastatic melanoma patients (N=17). Median age 62 years. 11 males, 6 females. 88.2% cutaneous melanoma, 5.9% mucosal melanoma, 5.9% unknown | - B cell density was significantly higher at the invasive margin compared to tumour centre, but no correlation to treatment response was observed. - No significant difference in B cell density was found between responders and non-responders. - No significant increase in B cell infiltration after treatment with ipilimumab. | No association |
| Onieva | High IGKC-Expressing Intratumoral Plasma Cells Predict Response to Immune Checkpoint Blockade | 2022 | Study of the gene expression and cellular levels of the tumour microenvironment that may predict response to checkpoint inhibitor immunotherapy. | Single cell RNA sequencing, multispectral immunofluorescence and machine learning | Discovery cohort of metastatic melanoma patients treated with nivolumab (N=21) (cutaneous N=16, uveal N=3, mucosal N=2). Validation cohort of metastatic melanoma patients treated with anti-PD1 (N=32). | - B cell related gene signatures such as BCR and immunoglobulin genes were differentially expressed in responding patients. - The expression of B cell-related genes was found to be associated with better progression-free and overall survival. - Naïve B cells, specifically IGK+ Naive B cells were significantly increased in good responders versus bad responders. - Multispectral IF showed higher B cell numbers in good responders, with an increased proportion of plasma cells in these patients. - A higher abundance and diversity of BCR signature was associated with better response to nivolumab. | **Positive association** |
| Pourmaleki | Tumour MHC Class I Expression Associates with Intralesional IL2 Response in Melanoma | 2022 | To study the immune microenvironment in melanoma lesions following intralesional IL-2 treatment, aiming to identify predictors of treatment response. | Multiplexed immunofluorescence, bulk RNA and DNA sequencing and immunohistochemistry | Patients with in-transit melanoma metastases (initial cohort N=7, validation cohort N=19). Patients treated with either intralesional or high-dose systemic IL2. | - Untreated lesions from high responding patients had significantly higher stromal B cell densities compared to non-responders. - B cell aggregates surrounded by T cells were observed significantly more frequently in highly responding patients. - B cell attractant CXCL13 was significantly upregulated in untreated lesions from highly responding patients. | **Positive association** |
| Quek | Single-cell spatial multiomics reveals tumour microenvironment vulnerabilities in cancer resistance to immunotherapy | 2024 | The study investigates the tumour microenvironment (TME) in metastatic melanoma patients treated with immunotherapy, focusing on the mechanisms of resistance and response to immune checkpoint inhibitors (ICIs). It explores the spatial and molecular features of the TME using single-cell and spatial multiomics approaches. | Single-cell RNA sequencing (scRNA-seq) CITE-seq: PhenoCycler (CODEX) Multimodal Integration Toolkit (MIT) Functional Enrichment Analysis Trajectory Analysis | Patients with metastatic melanoma (N=5). Patients were treated with anti-PD-1 (nivolumab or pembrolizumab) and/or anti-CTLA-4 (ipilimumab) therapy. One case of acquired resistance (MIA1), two cases of innate resistance (MIA2 and MIA11), and two cases of response. | - Lymphoid aggregates with high B cell signatures (e.g., TNFRSF13C, BLK, CD79A) were associated with improved progression-free survival (PFS) and overall survival (OS) in anti-PD-1-treated patients. - Lymphoid aggregates at the spatial level were identified not only in immunotherapy responding tumours but also in resistant tumours. | **Positive association** |
| Somasundaram | Tumour-associated B-cells induce tumour heterogeneity and therapy resistance | 2017 | Investigating the role of tumour-associated B cells in tumour heterogeneity and in resistance to BRAF and MEK inhibitors. | Co-culture assays, RNA sequencing, immunohistochemistry and flow cytometry | Patients with advanced, therapy-resistant melanoma (N=10). Patients with ≥4 metastatic sites (N=7), patients that received multiple previous systemic therapies (N=7) | - Tumour-associated B cells found to produce IGF-1, known to contribute to BRAF/MEK resistance. Co-cultures of melanoma cells and TABs showed significant resistance to BRAF inhibitors. - Transcript levels revealed significantly increased CD20 in therapy resistant samples compared to matched pre-treatment samples. - B cell-depletion showed promising anti-tumour activity in a small trial of therapy-resistant patients. A benefit in measurable disease as assessed by RECIST and ir-RC criteria was seen in 6 patients, such as partial response and stable disease. | **Negative association** |
| Valpione | Tumour infiltrating B cells discriminate checkpoint blockade-induced responses. | 2022 | Investigation into the role of B cell clonotypes, antigen presentation and intercellular communication within tumour-associated B cells and their influence on response to immune checkpoint blockade. | Single cell and bulk RNA sequencing, surface protein expression (REAP-Seq) | Primary cohort of metastatic melanoma patients (N=5). Validation cohort of pre-treatment melanoma biopsies (N=20) and pre-treatment melanoma samples from published datasets (N=120) | - Immunoglobulin genes (IgM, IgG and IgA) and B cell related genes (MZB1) were upregulated in pre-treatment biopsies from patients with controlled disease compared to progressive disease samples. Inverse correlation was observed between tumour purity and BCR abundance, and a direct correlation between tumour purity and top BCR clone prevalence was found. - High BCR clonotype abundance was associated with improved overall survival in patients receiving ICI immunotherapy. | **Positive association** |
| Varn | A B cell-derived gene expression signature associates with an immunologically active tumour microenvironment and response to immune checkpoint blockade therapy. | 2018 | Identification of B cell-related gene expressions in melanoma patients receiving anti-PD1 and anti-CTLA4 inhibitors that correlate to response efficacy. | RNA sequencing using publicly available dataset from TCGA | Melanoma patients receiving anti-PD1 inhibitors (N=28) and anti-CTLA4 inhibitors (N=42) | - Researchers found that patients with clinical benefit in response to anti-PD1 and anti-CTLA4 therapy had significantly higher memory B cell-like scores compared to those with no clinical benefit to therapy. - These patients with high memory B-like (MBL) score was associated with improved clinical benefit and overall survival. - Furthermore, in the anti-CTLA4 dataset BCR heavy chain abundance was significantly associated with clinical benefit to therapy and prolonged patient survival. | **Positive association** |
| Wu | CD20(+) CD22(+) ADAM28(+) B Cells in Tertiary Lymphoid Structures Promote Immunotherapy Response | 2022 | The study aimed to identify and characterize a subset of B cells, termed CD20+CD22+ADAM28+ B cells (BIR cells), within tertiary lymphoid structures (TLSs) and evaluate their role in promoting response to immune checkpoint inhibitor (ICI) therapy in cancer patients. | scRNA-seq and bulk RNA seq Immunofluorescence staining and immunohistochemistry. Flow cytometry and animal experiments. | scRNA-seq data from anti-PD-1 treated melanoma patients split into responders (N=17) and non-responders (N=31). Bulk RNA-seq data from anti-PD1 and anti-PD1/CTLA4 melanoma patients. | - BIR cells, a subset of memory B cells within TLSs, were found to enhance ICI therapy response by promoting anti-tumour immunity. BIR cells were enriched in ICI responders compared to non-responders. - Their presence correlated with prolonged survival and better clinical outcomes in multiple cancer types. - These cells exhibited a unique gene signature (e.g., CD20, CD22, ADAM28) and interacted with myeloid cells to sustain their function. | **Positive association** |
| Zhang | Comprehensive analysis of single cell and bulk RNA sequencing reveals the heterogeneity of melanoma tumour microenvironment and predicts the response of immunotherapy | 2024 | To interrogate single cell and bulk RNA sequencing datasets of melanoma patients to characterise tumour microenvironmental cells. | RNA sequencing using two publicly available datasets obtained from the Gene Expression Omnibus (GEO) database: GSE120575 and GSE115978. | N=79 patients included in the single cell dataset, N=6 patients receiving anti-PD1 or anti-CTLA4 for the bulk dataset | - A significantly higher proportion of B cells was reported in responders versus non responders to ICI immunotherapy. - Higher expression of B cells was found to be significantly associated with improved cumulative survival. | **Positive association** |

Supplementary Table 12. Studies investigating differential B cell responses when on treatment with different ICI targets

| Author | Title | Year | Assessment of the modulation of B cell signatures, or association between B cell signatures and response to different ICI therapies | ICI Comparison | Differential B cell responses between ICI therapies? |
| --- | --- | --- | --- | --- | --- |
| Das | Early B cell changes predict autoimmunity following combination immune checkpoint blockade | 2017 | - Circulating B cells decreased in patients following combination anti-PD1/CTLA4, but not in patients treated with anti-PD1/CTLA4 monotherapy. - Specific populations were proportionally increased on treatment, including class switched memory B cells and plasmablasts in the combination cohort and CD21lo B cells in the combination and anti-CTLA4 cohorts. - An early decline in B cell numbers after therapy directly correlated with onset of toxicity and associated with worse overall survival in patients with high grade toxicity. - Higher rates of irAEs were found in the combination cohort compared to the anti-PD1 and anti-CTLA4 monotherapy patients. | Combination anti-PD/anti-CTLA4 vs either anti-PD1 or anti-CTLA4 monotherapy | **Yes** |
| Gatto | Early Increase of Circulating Transitional B Cells and Autoantibodies to Joint-Related Proteins in Patients with Metastatic Melanoma Developing Checkpoint Inhibitor-Induced Inflammatory Arthritis | 2023 | - No difference in circulating B cells was observed during active ICI-IA (immune checkpoint inhibitor-induced inflammatory arthritis) between combination and monotherapy patients. - Proportion of on-treatment circulating CD19+ cells did not differ significantly between patients receiving stable anti-PD1 monotherapy or patients that received combination therapy prior to monotherapy. | Combination anti-PD/anti-CTLA4 vs either anti-PD1 or anti-CTLA4 monotherapy | **No** |
| Quek | Single-cell spatial multiomics reveals tumour microenvironment vulnerabilities in cancer resistance to immunotherapy | 2024 | - High expression of B cell genes was associated with significantly improved progression-free and overall survival versus no/low expression in patients treated with anti-PD1, but in patients treated with combination therapy (anti-PD1/CTLA4) no difference in prognosis was found. | Combination anti-PD/anti-CTLA4 anti-PD1 monotherapy | **Yes** |
| Varn | A B cell-derived gene expression signature associates with an immunologically active tumour microenvironment and response to immune checkpoint blockade therapy. | 2018 | - An MBL (memory B cell-like) score was used to predict response to anti-PD1 and anti-CTLA4 therapies. - For both cohorts of patients, treated with either anti-PD1 or anti-CTLA4 monotherapy, significantly higher MBL scores were observed in patients experiencing clinical benefit and improved probability of survival compared to patients that experienced no clinical benefit following treatment. - Reported that BCR heavy chain abundance was associated with clinical benefit and improved patient survival in the anti-CTLA4 dataset, but no significant association was found in the anti-PD1 dataset. | Anti-PD1 or anti-CTLA4 monotherapy | **No (MBL score)**  **Yes (BCR abundance)** |

Supplementary Table 13. Studies investigating antibody function and reactivity in melanoma

| Author | Title | Year of publication | Aims of Study | Techniques Used | Patient Demographics | Key Findings |
| --- | --- | --- | --- | --- | --- | --- |
| Cipponi | Neogenesis of lymphoid structures and antibody responses occur in human melanoma metastases. | 2012 | Study ectopic lymphoid structures, defined as lymphoid follicles comprising clusters of B lymphocytes and follicular dendritic cells (DC), associated with high endothelial venules (HEV) and clusters of T cells and mature DCs, in cutaneous metastases from melanoma patients. | Immunohistochemistry and qRT-PCR | Skin metastases from melanoma patients (N=29) | - B cell aggregates were detected in 14 of the 29 tumour samples, and plasma cells stained for CD138 were distributed more diffusely and irregularly outside the B-cell aggregates. - Dense B-cell clusters were associated with more centrally located cells expressing CD21, a complement receptor expressed selectively by FDC, and several follicles were found to host a small group of cells with nuclear expression of AID. - Identified 17 distinct repeated clones among 4 follicles and identified 8 clones in which a unique VDJ sequence was coupled to either of 2 different constant regions. - CSR was observed, including IgD to IgG1, IgG1 to IgG2, IgG1 to IgA1, and IgA1 to IgA2 transitions. |
| De Moel | Autoantibody development under treatment with immune- checkpoint inhibitors | 2019 | To study the impact of immune checkpoint inhibitors on autoantibody development in melanoma patients and association with immune related adverse events. | Immunofluorescence assays, fluorescence enzyme immunoassays, chemiluminescent immunoassays | Advanced melanoma patients (N=133) treated with ipilimumab. Mean age was 59 years, and 62% of patients were male | - Out of the 127 patients with pre- and post-treatment autoantibody data, 80% were autoantibody negative pre-treatment. 19.2% of patients from the autoantibody-negative cohort developed autoantibodies post-CTLA-4 treatment, predominantly anti-TPO and anti-TG. - A slight, but not significant association was found between the development of autoantibodies during ipilimumab treatment and irAEs. - In patients who received subsequent anti-PD1 therapy, a significant association was found between the development of thyroid autoantibodies while on ipilimumab and subsequent thyroid dysfunction under PD-1 blockade. - There was no significant association between the presence of a specific autoantibody and survival. |
| Diem | Immunoglobulin G and Subclasses as Potential Biomarkers in Metastatic Melanoma Patients Starting Checkpoint Inhibitor Treatment. | 2019 | This study evaluated immunoglobulin G subclasses in the serum of melanoma patients and their association with anti-tumour response. | Immunoturbidimetric methods | Metastatic melanoma patients (N=49). Monotherapy with nivolumab or pembrolizumab anti-PD1 (N=42), patients received combination nivolumab/ipilimumab (N=5), and ipilimumab monotherapy (N=2) | - Baseline levels of IgG2 were found to be significantly higher in responders vs non-responders to immunotherapy, with no differences observed in the levels of the other immunoglobulin isotypes or total IgG. - Progression-free survival was significantly better in patients with high levels of total IgG, IgG1, IgG2 and IgG3. Improved overall survival was associated with higher IgG2 levels. - No significant association was found between IgG levels and development of adverse events. |
| Ghosh | Lower baseline autoantibody levels are associated with immune-related adverse events from immune checkpoint inhibition | 2022 | To investigate the association between baseline autoantibodies and development of irAEs in patients with advanced melanoma | Autoantigen array, immunofluorescence, ELISA | Patients with unresectable stage III or IV melanoma (N=60) receiving combination ICI therapy (ipilimumab and nivolumab) | - Baseline and on-treatment plasma samples were tested against an array of 120 autoantigens commonly associated with autoimmune diseases. - Patients that developed irAEs were found to have lower levels of differentially expressed IgG autoantibodies at baseline compared to patients that did not develop irAEs. - A greater fold change in antibody concentration of IgG and IgM from baseline to 6 weeks correlated to irAE development. - No autoantibodies were found to be predictive of specific events. For ANA, RF and CCP autoantibodies specifically, no differences were found between seropositive and seronegative patients at baseline in irAE development, disease severity or survival. |
| Gilbert | Monitoring the systemic human memory B cell compartment of melanoma patients for anti-tumour IgG antibodies. | 2011 | Evaluate mature IgG antibody responses to melanoma from human peripheral blood B cells. | ELISA | Healthy volunteers (N=10) and melanoma patients (N=10) (N= 4 stage II, N = 4 stage III, and N = 2 stage IV) | - Significant fold increase in the mean reactivity of patient-derived antibody cultures to primary and metastatic melanoma cells compared to antibody cultures derived from healthy volunteers. - Analysis of antibody responses according to disease stage showed that patients with local (non-metastatic, stages I and II) disease had a significantly higher mean antibody response compared to those with confirmed metastatic disease (stages III and IV). |
| Karagiannis | Elevated IgG4 in patient circulation is associated with the risk of disease progression in melanoma | 2015 | The aim of the study was to investigate IgG4 as a predictor of risk for disease progression. | Luminex, immunohistochemistry and flow cytometry | The study involved metastatic melanoma patients (N=47) and healthy volunteers (N=24) | - Significantly elevated serum IgG4 levels (IgG4/IgG_total_) were detected in melanoma patients as compared to healthy controls. In early disease (Stages I–II) patients with SD (stable disease) during the study period displayed significantly lower serum levels of IgG4 as compared to patients who developed PD (progressive disease). - Kaplan-Meier curve evaluations revealed that the IgG4_high_ group displayed statistically significantly lower OS compared to the IgG4_low_ group. - The proportion of IgG4 cells in the peripheral blood B cell compartment was significantly elevated compared with that of healthy volunteers. |
| Karagiannis | IgG4 subclass antibodies impair antitumor immunity in melanoma | 2013 | Investigate the presence and functional implications of IgG4 in malignant melanoma. | *Ex vivo* stimulation assays, RNA and protein isolation from *in vitro* assays, western blots, ELISA, qtPCR, immunohistochemistry, Luminex, flow cytometry, image stream, ADCC/ADCP and antibody engineering | Patients with stage I-IV melanoma (N=57) and skin samples from healthy volunteers (N=18). PBMCs from healthy volunteers (N=8) | - CD22 + cell infiltrates were found in 8 out of 9 melanoma lesions examined. A significantly higher number of CD22+ B cells were found in primary melanoma tissue, and metastatic melanoma tissue compared to healthy skin. - Significantly higher IgG relative mRNA expression was found in primary melanoma tissue, and metastatic melanoma tissue compared to healthy skin, and when stratified by disease stage, significantly higher IgG relative mRNA expression found in stage IV disease compared to II. - B cells derived from metastatic melanoma lesions exhibited higher proportional IgG4 subclass production. From the patient-derived IgG1 and IgG4 antibodies, levels of IgG4 reactivity in 1 blood sample and 1 cutaneous metastasis was detected. - Patients with higher serum IgG4/IgG_total_ ratios had significantly lower survival rates than patients with lower IgG4/IgG_total_ serum ration. - Melanoma cells drive Th2 inflammation supporting B cells to produce IgG4 ex vivo. |
| Kessler | Serum IgG is associated with risk of melanoma in the Swedish Amoris study | 2019 | Investigate the role of the humoral immune system, specifically serum immunoglobulins (IgA, IgG, and IgM), in the development of melanoma, and whether pre-diagnostic levels of these immunoglobulins are associated with the risk of melanoma. | Serum levels of IgA, IgG, and IgM were measured using turbidimetric determination with a HITACHI 911 automatic analyser. | Individuals (N=29,876) aged 20 years or older from the AMORIS cohort. | - Higher pre-diagnostic serum IgG levels were associated with a reduced risk of melanoma, though the association was not statistically significant. - Compared to the reference IgG level, individuals with IgG levels ≥15.00 g/L had a hazard ratio (HR) of 0.60, indicating a non-significant inverse association. - In contrast, a positive association with risk of melanoma for those with IgG levels 6.10 g/L . |
| Mulder | Histopathological and immunological spectrum in response evaluation of talimogene laherparepvec treatment and correlation with durable response in patients with cutaneous melanoma | 2022 | Study the plasma cells present after treatment in cutaneous melanoma patients. | Immunohistochemistry and PCR | Patients with advanced melanoma (N=30) treated with T-VEC. Biopsies taken from 25 patients. | - Plasma cells were found in all biopsies. Plasma cell formation was observed at 3 months after T-VEC start. In 3 patients with multiple biopsies, further analysis revealed plasma cell infiltration of the IgG isotype, while IgM, IgA, and IgD were not or only incidentally detected. - There was a class switch of IgM to IgG with skewing to certain dominant Ig heavy chain clonotypes. - Plasma cells are probably a relevant feature in the mechanism of response but were not associated with durable response. |
| Saul | IgG subclass switching and clonal expansion in cutaneous melanoma and normal skin | 2016 | Study humoral immunity in cutaneous melanoma compared to normal skin. | IHC, RNA extraction qt-PCR, IgG sequence analysis and RNA seq | Melanoma patients (N=49) and healthy volunteers (N=24). For RNA seq analysis, 234 human skin and melanoma samples were extracted from the Gene Expression Omnibus (GEO) database (GSE7553, n = 58); (GSE46517, n = 93); (GSE8401, n = 83) and 384 human melanoma samples from The Cancer Genome Atlas (TCGA) database. | - IHC found CD22+ B cells in normal skin and melanoma tissue. CD22+ infiltrates were found in 37.6% of melanoma samples, with ~10% of melanomas featuring denser B cell infiltrating populations. - There is increased relative expression of CD20, CD22 and AID in metastatic compared with primary melanoma. Furthermore, a significant association between higher CD20 mRNA expression with longer patient survival. - From the molecular profiles of the mature IgG subclasses, 34% of the sequences detected from melanoma samples were IgG1, and 12% belonged to the IgG4 subclass. While normal skin samples displayed a similar distribution pattern, they had lower representation of IgG1/IgG_total_ sequences (24%). - These profiles were distinctly different to the IgG sequences from circulating B cells of patients with melanoma, in which IgG1 was the predominant subclass. |
